# Context-dependent effects of IL-2 rewire immunity into distinct cellular circuits

**DOI:** 10.1084/jem.20212391

**Published:** 2022-06-14

**Authors:** Carly E. Whyte, Kailash Singh, Oliver T. Burton, Meryem Aloulou, Lubna Kouser, Rafael Valente Veiga, Amy Dashwood, Hanneke Okkenhaug, Samira Benadda, Alena Moudra, Orian Bricard, Stephanie Lienart, Pascal Bielefeld, Carlos P. Roca, Francisco José Naranjo-Galindo, Félix Lombard-Vadnais, Steffie Junius, David Bending, Masahiro Ono, Tino Hochepied, Timotheus Y.F. Halim, Susan Schlenner, Sylvie Lesage, James Dooley, Adrian Liston

**Affiliations:** 1 Immunology Programme, The Babraham Institute, Cambridge, UK; 2 VIB Center for Brain and Disease Research, Vlaams Instituut voor Biotechnologie, Leuven, Belgium; 3 Department of Microbiology, Immunology and Transplantation, KU Leuven—University of Leuven, Leuven, Belgium; 4 Toulouse Institute for Infectious and Inflammatory Diseases (Infinity), Centre national de la recherche scientifique U5051, Institut national de la santé et de la recherche médicale U1291, University of Toulouse III, Toulouse, France; 5 Imaging Facility, The Babraham Institute, Cambridge, UK; 6 Centre de Recherche Sur L’inflammation, Centre national de la recherche scientifique ERL8252, Institut national de la santé et de la recherche médicale U1149, Université de Paris, Paris, France; 7 Department of Microbiology and Immunology, McGill University, Montréal, Quebec, Canada; 8 Department of Immunology-Oncology, Maisonneuve-Rosemont Hospital, Montréal, Quebec, Canada; 9 Institute of Immunology and Immunotherapy, College of Medical and Dental Sciences, University of Birmingham, Birmingham, UK; 10 Department of Biomedical Molecular Biology, Ghent University, Ghent, Belgium; 11 VIB Center for Inflammation Research, Vlaams Instituut voor Biotechnologie, Ghent, Belgium; 12 Cancer Research UK Cambridge Institute, University of Cambridge, Cambridge, UK; 13 Département de Microbiologie, Infectiologie et Immunologie, Université de Montréal, Montréal, Quebec, Canada; 14 Department of Life Sciences, Imperial College London, London, UK

## Abstract

Interleukin 2 (IL-2) is a key homeostatic cytokine, with therapeutic applications in both immunogenic and tolerogenic immune modulation. Clinical use has been hampered by pleiotropic functionality and widespread receptor expression, with unexpected adverse events. Here, we developed a novel mouse strain to divert IL-2 production, allowing identification of contextual outcomes. Network analysis identified priority access for Tregs and a competitive fitness cost of IL-2 production among both Tregs and conventional CD4 T cells. CD8 T and NK cells, by contrast, exhibited a preference for autocrine IL-2 production. IL-2 sourced from dendritic cells amplified Tregs, whereas IL-2 produced by B cells induced two context-dependent circuits: dramatic expansion of CD8^+^ Tregs and ILC2 cells, the latter driving a downstream, IL-5–mediated, eosinophilic circuit. The source-specific effects demonstrate the contextual influence of IL-2 function and potentially explain adverse effects observed during clinical trials. Targeted IL-2 production therefore has the potential to amplify or quench particular circuits in the IL-2 network, based on clinical desirability.

## Teaser

Network analysis of IL-2 identifies a context-dependent hierarchy of consumption and response. IL-2 sourced from different leukocytes drives different circuits of responding cells, independent of concentration. Targeting IL-2 localization may therefore allow fine-tuning of therapeutic responses.

## Graphical Abstract

**Figure:**
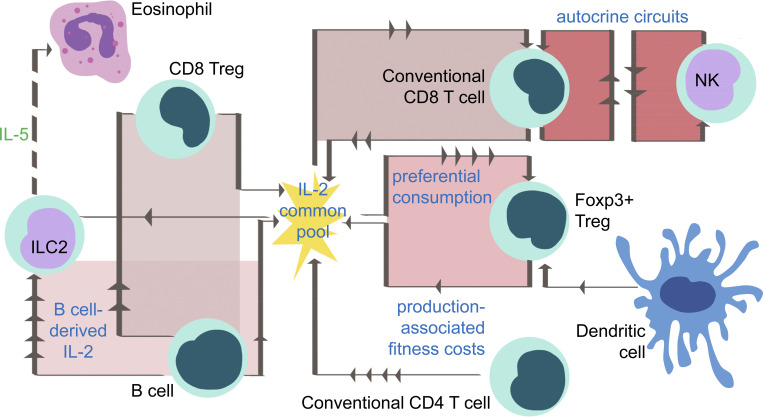

## Introduction

IL-2 is one of the key homeostatic cytokines controlling the immune system (Malek, 2008) and was among the first cytokines to be discovered (Gillis et al., 1978). Our understanding of IL-2 has shifted from a broad-utility T cell growth factor into a complex cytokine impacting T cell differentiation, proliferation, and survival, with a profound effect on regulatory T cell (Treg) “fitness” (Fontenot et al., 2005). Individual cellular circuits of IL-2 signaling have been identified and characterized in depth. Arguably, the most important of these involves the early production of IL-2 by activated CD4 T cells (Amado et al., 2013), which in turn drives the expansion of Treg numbers via altering the proliferation and apoptotic kinetics (Obata et al., 2014; Pierson et al., 2013; Shi et al., 2018). While this creates a closed circuit, with negative feedback properties (Liston and Gray, 2014; Smith and Popmihajlov, 2008), this network can be rewired upon a change of context, such as driving a positive feedback loop of inflammatory CD8 T cell activation (Humblet-Baron et al., 2019; Humblet-Baron et al., 2016). Here the rewiring is based in part on the expression of the high-affinity (*K*_*d*_ ≈ 10^−11^ M) trimeric receptor of CD25, CD122, and CD132, expressed constitutively by Tregs and upon activation in CD8 T cells. The preferential capture of IL-2 by CD25 provides a competitive advantage over cells (mostly naive CD8 T cells and natural killer [NK] cells) that largely express the intermediate-affinity (*K*_*d*_ ≈ 10^−9^ M) dimer of CD122 and CD132 (Spangler et al., 2015). The system is further complicated by the CD122/CD132 dimer acting as a high-affinity receptor for IL-15/IL-15RA complexes. Despite binding with the same structural geometry as IL-2 (Ring et al., 2012), IL-15 signaling diverges from IL-2 signaling, in part because of temporal binding differences (Cornish et al., 2006). Nonetheless, the shared receptors allow high-dose IL-2 to mimic aspects of IL-15 signaling. The existence of alternative affinity receptors, dynamically regulated in quantity and expressed on multiple cell types, demonstrates the potential plethora of cellular circuits that could be controlled by IL-2.

A complete understanding of the network effects of IL-2 on immune homeostasis will need to go beyond the identification of pairwise circuits. Homeostatic networks can be defined based on controlled variables and regulated variables, with controllers acting on plants to stabilize the system (Kotas and Medzhitov, 2015). Using these definitions, activated CD4 T cells can be considered plants, producing the controlled variable IL-2 to regulate Treg numbers. However, in a biological system as complex as the immune system, the distinction between these homeostatic components is obscured by multiple layers of regulation, interconnection between variables, conditional dependence of signals, and cell types simultaneously acting in multiple roles (Kotas and Medzhitov, 2015). Tregs, for example, can be considered both a regulated variable, stabilized by IL-2, and a controller that acts on CD4 T cells to tune IL-2 expression. The broader the set of cells and potential interactions considered, the more complex the potential effects of each network component can be. For signaling components with high biological potency, such as IL-2, a more complete understanding of the network may depend on top-down measurements of network-level perturbations, rather than bottom-up construction of the network from the building blocks of defined circuits.

Moving toward a more complete understanding of the IL-2 network does not serve solely as a proof-of-principle for dissecting the complex biology of pleiotropic cytokines. IL-2 is also an actively investigated therapeutic drug, the subject of hundreds of ongoing clinical trials. This treatment context makes understanding overexpression and gain-of-function effects physiologically relevant to patients. The importance of the contextual aspects of the IL-2 network lies in its diametrically opposed clinical uses. IL-2 has been adopted for its ability to distort immunity toward either an immunostimulatory or immunosuppressive state, based on the opposing targets of conventional T cells (Tconv) and Tregs, respectively. Initially Food and Drug Administration−approved for treatment of metastatic renal cell carcinoma and melanoma in the 1990s (Rosenberg, 2014), before the resurgence of Treg biology, high doses of IL-2 are used as stimulatory immunotherapy. The key target of this approach is generally antitumor CD8^+^ T cell responses, although trials are also underway to enhance infectious immunity (Pol et al., 2020). Conversely, the identification of CD25^+^ Tregs led to the design of low-dose IL-2 trials, aimed at enhancing Treg numbers and function. These trials aimed to suppress pathological and autoimmune responses, in diseases ranging from type 1 diabetes to graft-versus-host disease (Hartemann et al., 2013; Koreth et al., 2011; Matsuoka et al., 2013).

Despite promising results in a proportion of patients, the therapeutic efficacy of IL-2 has been hampered by the pleiotropic effects on diverse cell types. Toxicity is often observed, with a vast array of side effects reported in patients, including vascular leak syndrome, hypotension, and end-organ dysfunction, often leading to discontinuation of treatment or death (Dutcher et al., 2014). These toxicities are particularly apparent with the high doses of IL-2 that are required to stimulate CD8 T cell proliferation. A potential explanation for the complexities in outcome following IL-2 treatment is the lack of specificity, with an active area of research aiming to improve therapeutic IL-2 by altering its affinity for its receptors (Abbas et al., 2018; Boyman et al., 2006; Letourneau et al., 2010). Alternatively, contextual effects, arising from conditional dependence of signals, may explain the unexpected clinical effects. Sufficient understanding of the IL-2 homeostatic network, however, is first required to determine whether contextual signaling is involved.

The detailed study of the responsiveness to IL-2 of individual cell types has focused on exogenous provision to the desired therapeutic targets of Treg and CD8 T cells. A systematic network analysis of IL-2 sources and effects has been lacking. For successful utilization of IL-2 in the clinic, this immunological network understanding is critical. Here we developed a novel mouse strain for dissecting IL-2 network effects and found that the biological effect of IL-2 differs markedly based on the cellular source. Even in the most studied axes of the IL-2 network, the responsiveness of Tregs and CD8, new modalities were determined, with preferential responsiveness by Treg to IL-2 delivered in trans and by CD8 to IL-2 delivered in cis. Alternative contexts for IL-2 provision resulted in new IL-2–dependent biological circuits arising, the most notable being the dramatic expansion of eosinophils and CD8 Tregs following local IL-2 delivery by B cells. These results have profound implications for the clinical delivery of ectopic IL-2, with the potential to tailor immunological outcomes by altering the context of delivery rather than manipulating molecular characteristics.

## Results

### A genetic switch for rewiring of the IL-2 production network

**Figure 1.**
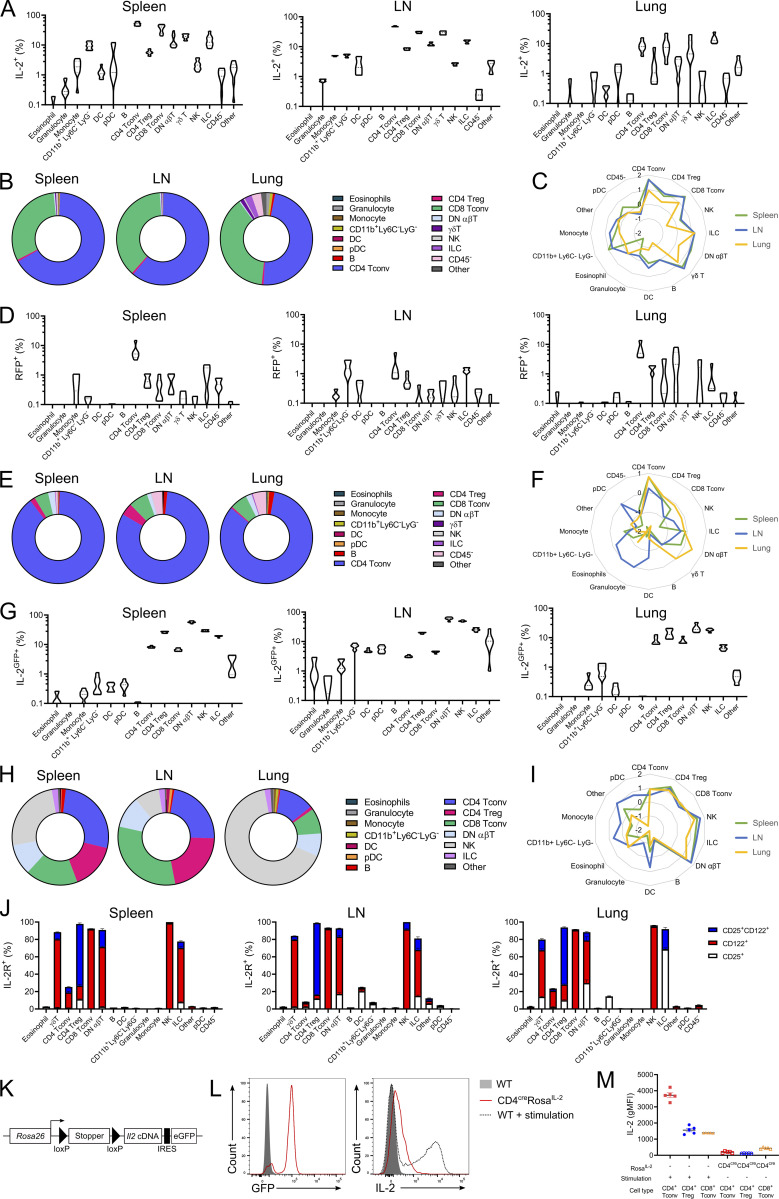
**A complex production landscape for IL-2 in the homeostatic immune system. (A)** IL-2 expression in spleen, LN, and lung from WT cells stimulated ex vivo with PdBU/ionomycin for 4 h. *n* = 6–9. pDC, plasmacytoid DC. **(B)** Frequency of each annotated cell type among the total IL-2^+^ population following ex vivo stimulation. **(C)** Radar plot of frequency (log_10_) of IL-2^+^ population following ex vivo stimulation. **(D)** RFP expression in spleen, LN, and lung from *Il2*^cre^*Rosa*^*RFP*^ mice. *n* = 6. **(E)** Frequency of each annotated cell type among the total RFP^+^ population from *Il2*^cre^*Rosa*^*RFP*^ mice. **(F)** Radar plot of frequency (log_10_) of RFP^+^ population. **(G)** IL-2 expression in spleen, LN, and lung of IL-2^GFP^ mice. *n* = 6. **(H)** Frequency of each annotated cell type among the total GFP^+^ population from IL-2^GFP^ mice. **(I)** Radar plot of frequency (log_10_) of GFP^+^ population. **(J)** Expression of low-affinity (CD25^+^), intermediate-affinity (CD122^+^), or high-affinity (CD25^+^CD122^+^) IL-2 receptors in spleen, LN, and lung WT cells. *n* = 10. **(K)** Genetic construct of *Rosa*^*IL-2*^ mice. **(L)** Representative histogram of GFP and IL-2 expression in CD4^+^ Tconv from WT and CD4^cre^Rosa^IL-2^ mice incubated for 4 h in the presence of BrefA or WT CD4^+^ Tconv stimulated for 4 h with PdBU/ionomycin/BrefA. **(M)** Geometric mean fluorescence intensity (gMFI) of IL-2 expression from ex vivo–stimulated WT mice or nonstimulated CD4^cre^*Rosa*^*IL-2*^ mice. *n* = 5. Data are representative of (A, B, G–I, L, and M) or pooled from (C and D) at least two independent experiments.

To define the sources of IL-2 production in the homeostatic system, we developed a highly sensitive flow cytometry protocol optimized for the detection of IL-2 production. As previously reported, CD4 Tconv cells demonstrated the highest potential for IL-2 production among the stimulated leukocyte lineages in the spleen, LN, and lung tissue (Fig. 1, A–C). IL-2 production was also reliably detected in Foxp3^+^ Tregs, CD8 T cells, peripheral double-negative (DN) T cells, γδ T cells, and innate lymphoid cells (ILCs), expanding the potential sources of IL-2 to lineages previously thought to be silenced for this cytokine (Fig. 1 A). Based on the frequency of these lineages, CD4 Tconv cells comprised ∼65% of all IL-2–producing cells in the spleen and LN, with CD8 T cells much of the remainder (Fig. 1 B). As this system depends on stimulated production, we also assessed sources of IL-2 production using an IL-2 fate-mapping system (*Il2*^*Cre*^ *Rosa*^*RFP*^ mice; Fig. 1, D–F) and *Il2*^*GFP*^ reporter mice (Fig. 1, G–I). Both systems confirmed the diversity of IL-2 sources; indeed, a higher fraction of IL-2 production came from cells other than CD4 Tconv cells in these stimulation-independent assays. Notably, the contribution of nontraditional cell types to IL-2 production was greater in nonlymphoid tissues such as the lung (Fig. 1, D–I). Corresponding mapping of IL-2 receptor expression identified Tregs as the dominant high-affinity receptor expressers, diverse lineages expressing the intermediate receptor (γδ T cells, CD8 T cells, peripheral DN T cells, NK cells, and ILCs in secondary lymphoid tissues) and few lineages expressing the low-affinity receptor, mainly dendritic cells (DCs) and lung ILCs (Fig. 1 J). Cells expressing the intermediate receptor complex (CD122^+^CD132^+^) can also respond to IL-15, when presented in trans with the IL-15RA, or, with lower affinity, to IL-15 directly. To perturb this IL-2 production network, we developed a genetic switch for IL-2 expression. Using the constitutive *Rosa26* promoter and a floxed-stop expression system (Fig. 1 K), we created a system in which Cre expression would induce cell lineage–specific IL-2 expression. The weak endogenous *Rosa26* promoter was used to limit the extent of IL-2 overexpression. Transgenic IL-2 expression in this system on a per-cell basis was ∼5% of the expression level of native IL-2 production by CD4 Tconv cells (Fig. 1, L and M), although the aggregate effect of constitutive production in cells that do not express IL-2 in the baseline state results in a net overexpression of IL-2, the extent varying based on the experimental conditions. Together, these results identified a potentially complex IL-2 production network and the ability to perturb that network in a directed fashion.

### Inverted consequences of IL-2 production and response in CD4 and CD8 T cells drive differential network effects

**Figure 2.**
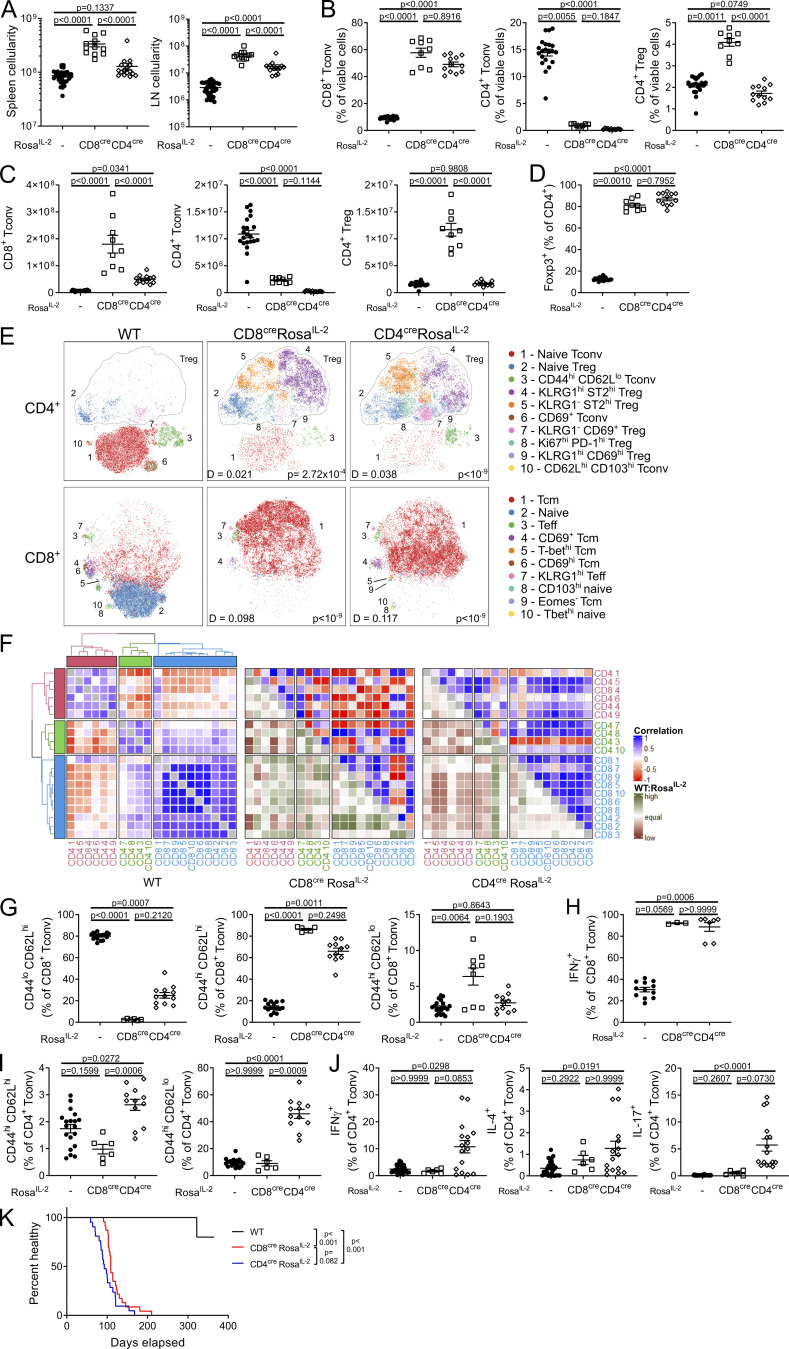
**Chronic expansion of CD8**^**+**^ **Tconv cells follows IL-2 dysregulation despite heightened Treg representation. (A)** Cellularity of spleen and LN of 4–6-wk-old CD8^cre^ *Rosa*^*IL-2*^, CD4^cre^ Rosa^IL-2^, or littermate controls. *n* = 12–34. **(B and C)** Frequency (B) and number (C) of splenic CD8^+^ Tconv, CD4^+^ Tconv, and CD4^+^ Tregs. *n* = 9–21. **(D)** Frequency of Foxp3^+^ cells among total CD4^+^ T cells. *n* = 9–21. **(E)** tSNE representation of high-parameter flow cytometry data from splenic CD4^+^ (top) and CD8^+^ (bottom) T cells. FlowSOM clusters annotated based on differential expression of key markers. D value represents cross-entropy distance between samples. **(F)** Correlation between clusters in E. WT:Rosa^IL-2^ indicates whether correlation is higher or lower in WT relative to CD8^cre^ or CD4^cre^ Rosa^IL-2^. **(G)** Frequency of naive, central memory (CD44^hi^ CD62L^hi^), or effector (CD44^hi^ CD62L^lo^) CD8^+^ Tconv. *n* = 6–19. **(H)** IFNγ expression by CD8^+^ Tconv. *n* = 3–12. **(I)** Frequency of central memory (CD44^hi^ CD62L^hi^) or effector (CD44^hi^ CD62L^lo^) CD4^+^ Tconv. *n* = 6–19. **(J)** Cytokine production from CD4^+^ Tconv. *n* = 6–32. **(K)** Healthy survival analysis, indicating onset of moderate symptoms. *n* = 5–23. Data representative of (H) or pooled from (A–G and I–K) at least two independent experiments. Significance was tested by one-way ANOVA (A and C), Kruskal–Wallis test (B and G–J), multiple Kolmogorov–Smirnov tests with Holm correction (E), or Mantel–Cox log-rank test (K).

**Figure S1.**
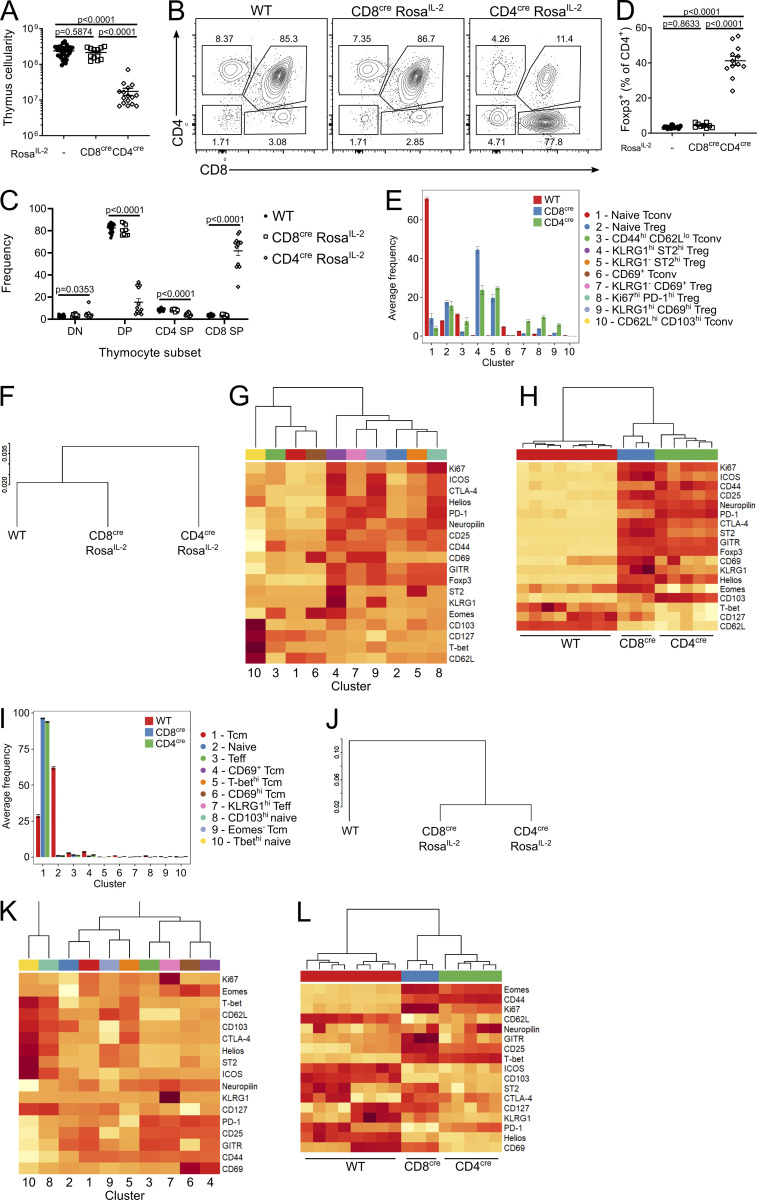
**T cell–driven IL-2 induces large-scale phenotypic shifts in CD4 and CD8 T cells. (A)** Thymic cellularity of 4–6-wk-old CD8^cre^ *Rosa*^*IL-2*^, CD4^cre^ *Rosa*^*IL-2*^, or littermate controls. *n* = 12–34. **(B and C)** Representative gating (B) and frequency (C) of thymocyte subsets. *n* = 9–21. DP, double-positive; SP, single-positive. **(D)** Frequency of Foxp3^+^ cells among total CD4 T cells. *n* = 9–21. **(E)** CD4 T cell data from 4–6-wk-old CD8^cre^ Rosa^IL2^, CD4^cre^ Rosa^IL2^, or littermate controls from Fig. 2 E. Average frequency of each cluster per mouse. **(F)** Dendrogram showing comparative similarity calculated using cross-entropy distributions from tSNE. **(G)** Heatmap showing differential marker expression in annotated FlowSOM clusters. **(H)** Heatmap showing differential marker expression in total CD4^+^ T cells from different strains, showing mouse replicates. **(I)** CD8 T cell data from 4–6-wk-old CD8^cre^ Rosa^IL2^, CD4^cre^ Rosa^IL2^, or littermate controls from Fig. 2 E. Average frequency of each cluster per mouse. **(J)** Dendrogram showing comparative similarity calculated using cross-entropy distributions from tSNE. **(K)** Heatmap of differential marker expression in annotated FlowSOM clusters. **(L)** Heatmap of differential marker expression in total CD8^+^ T cells from different strains, showing mouse replicates. Data pooled from two or more independent experiments. Significance was tested by one-way ANOVA (A, C, and D).

The development of a Cre-inducible IL-2 system allowed us to constitutively drive IL-2 within the major IL-2–producing lineages. We first crossed the *Rosa*^*IL-2*^ allele to CD4-Cre, active in both CD4 and CD8 T cells from the late DN stage of thymic development, and the peripheral enhancer of CD8-Cre, active only in peripheral CD8 T cells. While the level of IL-2 produced by the genetic driver was much lower than the physiological capacity of these cells (Fig. 1, L and M), the system allows for constitutive expression, independent of antigen-mediated stimulation. Expression of additional constitutive IL-2 by CD8 T cells dramatically increased the cellularity of the spleen and LN (Fig. 2 A), largely through expansion of the number of CD8 T cells and, to a lesser extent, Tregs (Fig. 2, B and C). Use of the CD4-Cre transgene surprisingly had a lower impact (Fig. 2, A–C), likely due to reduced thymopoiesis (Fig. S1, A–D). Notably, both the relative and absolute numbers of CD4 Tconv cells collapsed following the provision of IL-2 either in trans, by CD8 T cells, or both in cis and in trans (Fig. 2, B and C). This effect may have been mediated through a detrimental impact of IL-2 on CD4 Tconv cells or through a downstream effect of the large increase in Tregs, expanded to 70–90% of all CD4 T cells (Fig. 2 D). At a phenotypic level, CD4 and CD8 T cells were substantially altered by the IL-2 provision (Fig. 2, E and F; and Fig. S1, E–L). CD8 T cells suffered a relative loss of naive T cells (Fig. 2 G), driven almost entirely by a substantial increase in IFNγ-producing central memory T cells (Tcm cells; Fig. 2, G and H). The collapse in CD4 T cell numbers was observed in both the CD4-Cre and CD8-Cre drivers. In the CD4-Cre driver only, this included a relative increase in activated CD4 T cells (Fig. 2 I) and an expansion of T helper 1 (Th1) and Th17 cells (Fig. 2, I and J), although this was offset by the decrease in absolute number of CD4 T cells (Fig. 2 C). With both drivers, mice developed manifestations of stress requiring euthanasia at ∼4 mo of age (Fig. 2 K), with large-scale lymphoproliferation evident (Fig. 2 A). Together, these results demonstrate that restraint in T cell production of IL-2 is required for long-term health, despite the increase in Treg numbers that accompanies constitutive expression.

**Figure 3.**
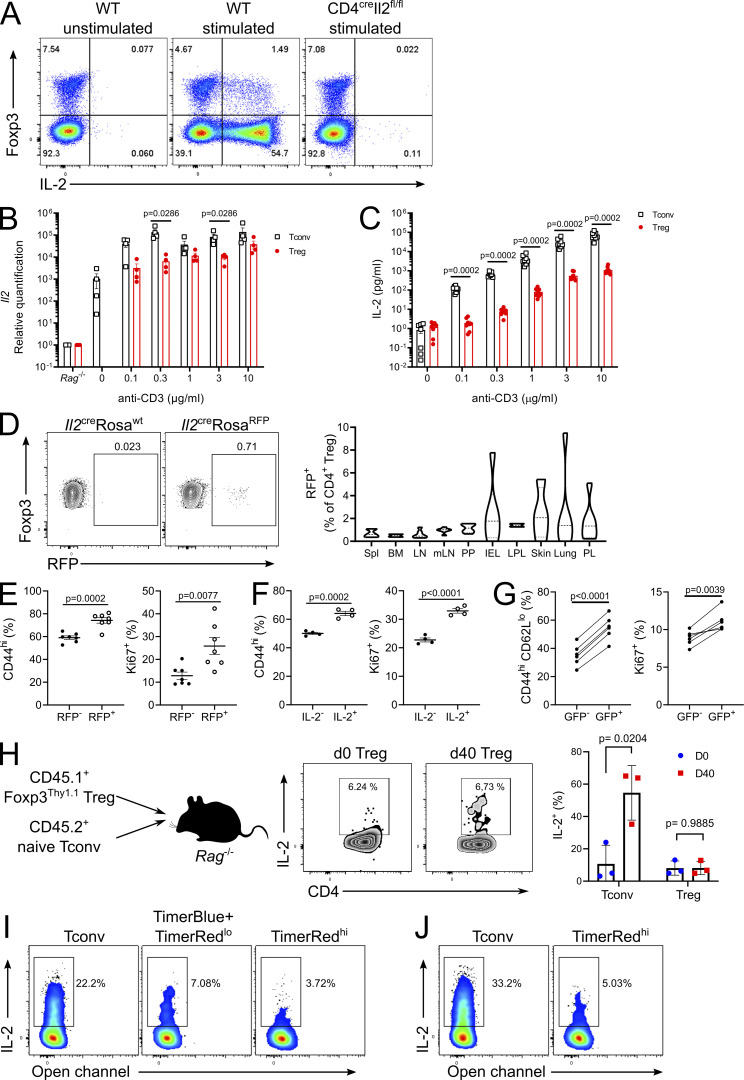
**Stable Tregs can produce IL-2 in vivo. (A)** Representative flow plots of IL-2 expression in CD4^+^ T cells from CD4^wt^*Il2*^fl/fl^ (WT) and CD4^cre^*Il2*^fl/fl^ mice. **(B)** *Il2* transcript in CD4^+^ Tconv and CD4^+^ Treg cells stimulated in vitro with anti-CD3. **(C)** IL-2 in supernatant of CD4^+^ Tconv and CD4^+^ Treg cells stimulated in vitro with anti-CD3. **(D)** IL-2 expression by CD4^+^ Tregs from *Il2*^cre^Rosa^RFP^ mice. *n* = 6. IEL, intraepithelial lymphocyte; LPL, lamina propria lymphocyte; mLN, mesenteric LN; PL, peritoneal lavage; PP, Peyer’s patch; Spl, spleen. **(E)** Frequency of CD44 and Ki67 expression in Tregs from *Il2*^cre^Rosa^RFP^ mice. *n* = 7. **(F)** Frequency of CD44 and Ki67 expression in IL-2^−^ and IL-2^+^ Tregs from ex vivo stimulation of WT cells. *n* = 4. **(G)** Frequency of CD44^hi^CD62L^lo^ and Ki67 expression in Tregs from *Il2*^GFP^ mice. *n* = 6. **(H)** IL-2 expression by CD4^+^ Tconv and CD4^+^ Tregs, 40 d following adoptive transfer into Rag^−/−^ mice. *n* = 3. **(I and J)** Representative expression of IL-2 by CD4^+^ T cells sorted from thymus (I) and spleen (J) of *Foxp3*-Tocky mice and stimulated ex vivo. Data are pooled from (D) or representative of (A–C, E, F, I, and J) at least two independent experiments. Significance was tested by Mann–Whitney *U* test (B and C), paired *t* test (E–G), or unpaired *t* test (H).

The identification of a small population of IL-2–producing Tregs (Figs. 1 A and 3 A) demonstrates that the reported *Il2* silencing through chromatin inaccessibility (Hemmers et al., 2019; Popmihajlov and Smith, 2008) is incomplete. Stimulation of Treg, sorted to a purity of >99%, with anti-CD3 in vitro confirmed that Tregs can produce *Il2* transcript and secrete IL-2 protein, although to a lesser extent than CD4 Tconv cells (Fig. 3, B and C). Only ∼1% of Tregs had a fate memory of IL-2 production in lymphoid organs, but substantially higher numbers were observed in nonlymphoid environmental-interface tissues (Fig. 3 D). Differences in IL-2–competent Treg population frequency when using native IL-2 detection, Cre-mediated IL-2 fate-mapping, and the *Il2*^*GFP*^ reporter likely reflect a combination of genomic contextual changes in the genetic constructs and disparities in tracing system, with fate-mapping requiring sufficient pulsed expression while the GFP reporter can read out accumulated low-level expression. Phenotypic comparison of IL-2 fate-mapped (Fig. 3 E), stimulated IL-2 expressers (Fig. 3 F), and unstimulated IL-2 reporter expressing Tregs from *Il2*^GFP^ mice (Fig. 3 G) demonstrated that the IL-2–producing Tregs were more likely to be activated and proliferating, suggesting that loss of locus silencing is associated with stimulation. To determine whether this phenomenon was restricted to newly formed Tregs or was a result of transient Foxp3 expression by effector T (Teff) cells, we purified CD4^+^Foxp3^+^ cells, transferred the cells into a lymphopenic environment, and assessed for IL-2 expression in Foxp3^+^ cells 40 d after transfer. Similar levels of IL-2 expression were observed in ex vivo Foxp3^+^ cells and posttransfer Foxp3^+^ cells, indicating that IL-2 is produced by a subset of T cells with long-term stable Foxp3 expression (Fig. 3 H).

As an independent approach, we used *Foxp3*-Tocky mice, in which *Foxp3* transcription is tracked by a Timer protein that has a short maturation time from blue to red fluorescence (Bending et al., 2018a; Bending et al., 2018b). The system allows the distinction between cells that recently initiated *Foxp3* expression (TimerBlue^+^TimerRed^lo^), such as recently converted Tregs or Teff cells with transient Foxp3 expression, versus cells with stable Foxp3 expression (TimerRed^hi^). Using this system, cells that had recently initiated *Foxp3* expression in the thymus (TimerBlue^+^TimerRed^lo^) had higher expression of IL-2 than established Tregs (TimerRed^hi^; Fig. 3 I), suggesting a gradual but incomplete repression of the *Il2* locus with Foxp3 induction in thymic Tregs. Consistent with our prior results, a fraction of splenic TimerRed^hi^ Tregs, with established and stable Foxp3 expression, also expressed IL-2 upon stimulation (Fig. 3 J). Together, these independent approaches demonstrate competency for low levels of IL-2 expression, protein production, and secretion in Foxp3^+^ cells. The phenotypic similarity between IL-2–producing and nonproducing Foxp3^+^ cells and the equivalent production in cells with long-term stable Foxp3 expression are most consistent with the incomplete silencing of the *Il2* locus by Tregs, with a partially preserved IL-2 production and secretion capacity. This incomplete silencing explains recent findings that *Il2*^*−/−*^ Tregs have poorer survival upon lymphopenic transfer (Chawla et al., 2020).

**Figure 4.**
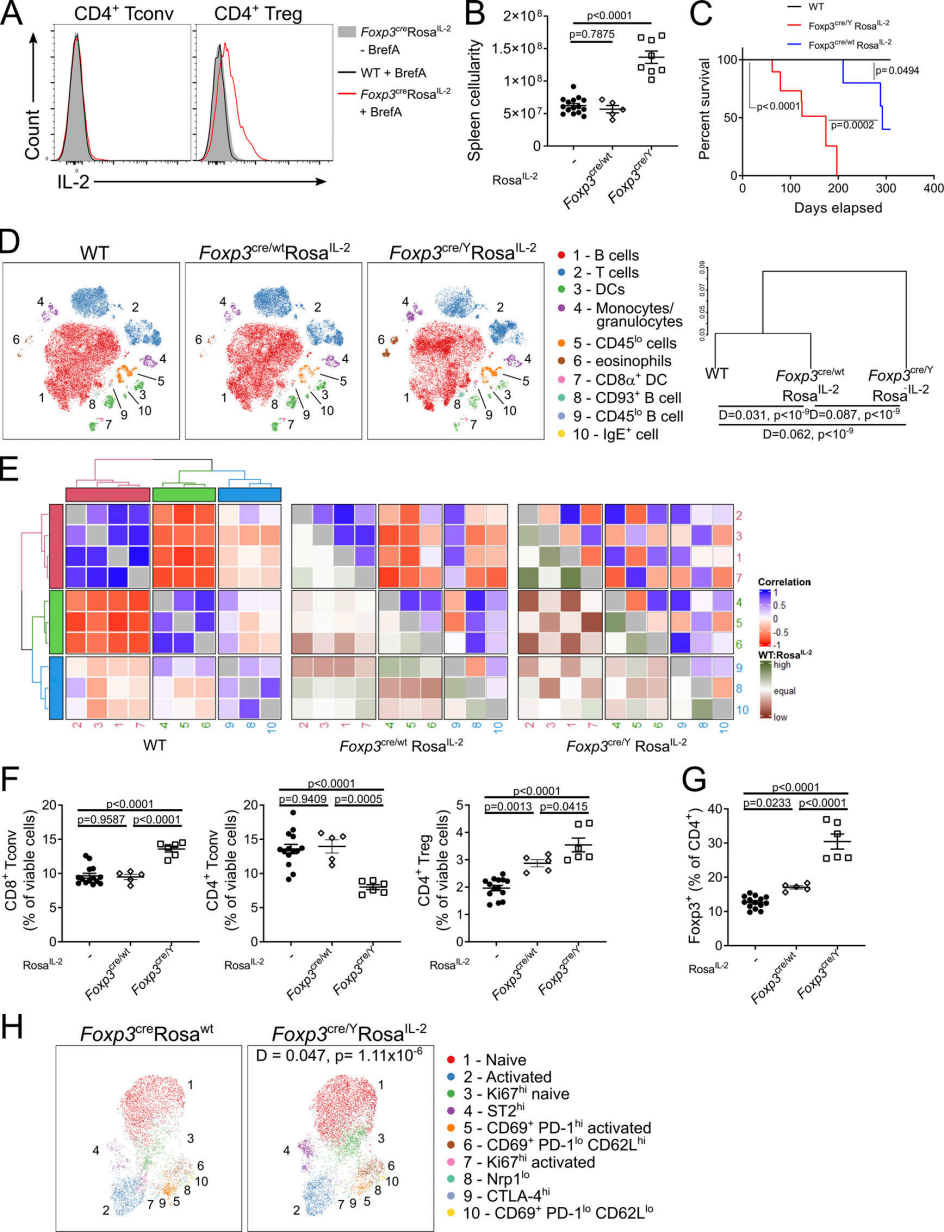
**Treg self-sufficiency for IL-2 drives dose-dependent expansion. (A)** Representative histograms of IL-2 expression in WT or *Foxp3*^cre^Rosa^IL-2^ splenocytes incubated with BrefA for 4 h to prevent cytokine secretion. **(B)** Splenic cellularity of *Foxp3*^cre/wt^*Rosa*^*IL-2*^, *Foxp3*^cre/Y^*Rosa*^*IL-2*^, or littermate controls. *n* = 5–15. **(C)** Survival analysis. *n* = 11–23. **(D)** tSNE representation of high-parameter flow cytometry data from viable splenocytes (left). FlowSOM clusters annotated based on differential expression of key markers. Dendrogram showing comparative similarity calculated using cross-entropy distributions from tSNE (right). **(E)** Correlation between clusters in D. WT:Rosa^IL-2^ indicates whether correlation is higher or lower in WT relative to *Foxp3*^cre/wt^ or *Foxp3*^cre/Y^ Rosa^IL-2^. **(F)** Frequency of splenic CD8^+^ Tconv, CD4^+^ Tconv, and CD4^+^ Treg cells. *n* = 5–15. **(G)** Frequency of Foxp3^+^ cells among total CD4^+^ T cells. *n* = 5–15. **(H)** Uniform Manifold Approximation and Projection (UMAP) representation of high-parameter flow cytometry data of *Foxp3*^cre^-expressing Tregs from *Foxp3*^cre/wt^Rosa^wt^ or *Foxp3*^cre/wt^Rosa^IL-2^ mice. FlowSOM clusters annotated based on differential expression of key markers. D value represents cross-entropy distance between samples. Data are pooled from at least two independent experiments. Significance was tested by one-way ANOVA (B, F, and G), Mantel–Cox log-rank test (C), multiple Kolmogorov–Smirnov test with Holm correction (D), or two-sample Kolmogorov–Smirnov test (H).

**Figure S2.**
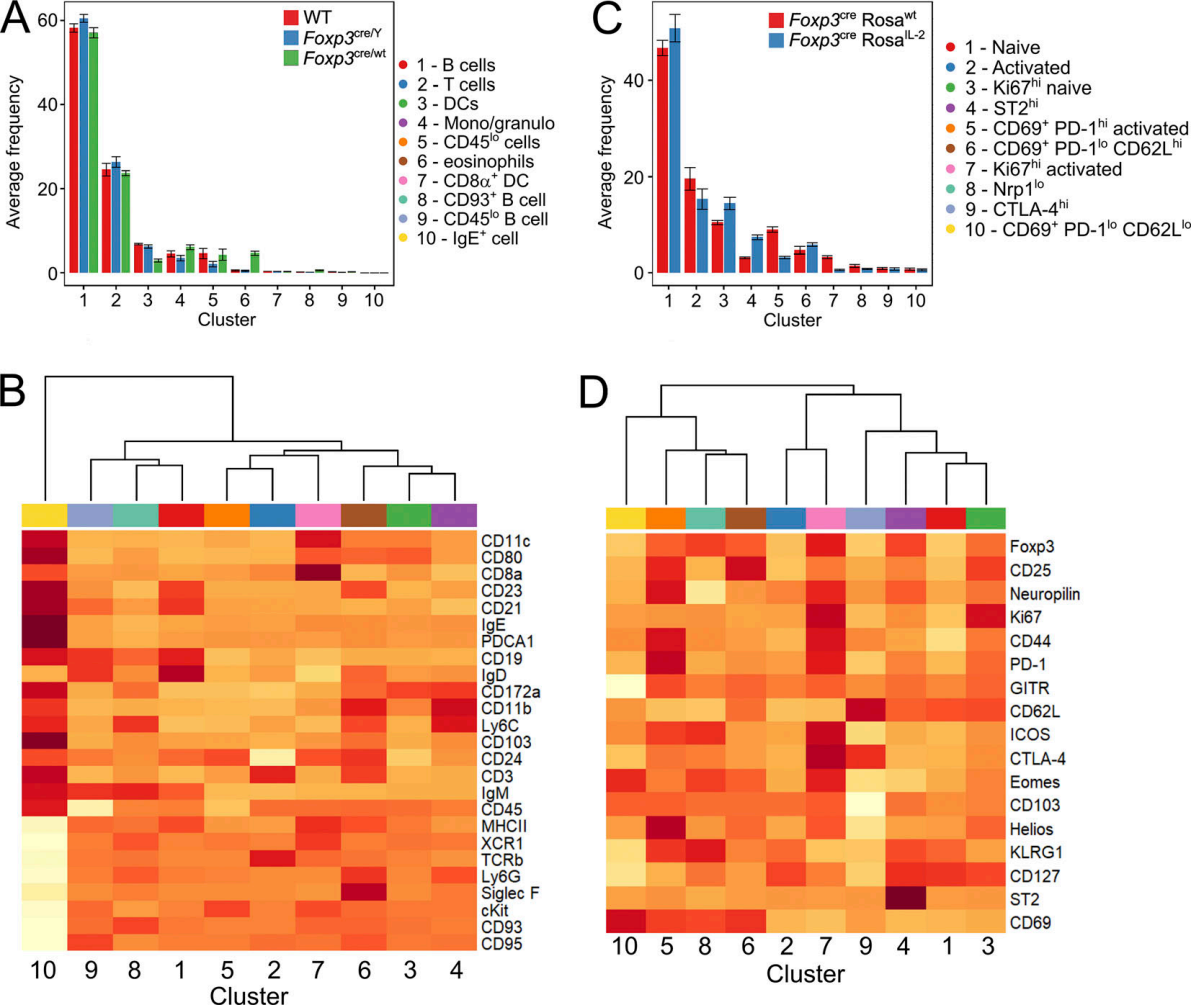
**IL-2 self-sufficiency drives Treg expansion without altering subset composition.** Splenocyte data from 4–6-wk-old *Foxp3*^cre/wt^*Rosa*^*IL-2*^, *Foxp3*^cre^*Rosa*^*IL-2*^, or littermate controls from Fig. 3 G. *n* = 5–15. **(A)** Average frequency of each cluster per mouse. **(B)** Heatmap showing differential marker expression in annotated FlowSOM clusters. **(C)** Average frequency of each Treg subcluster per mouse, from Fig. 3 J. **(D)** Heatmap showing differential marker expression in annotated FlowSOM Treg subclusters.

Having established IL-2 expression by Tregs, we intercrossed the *Rosa*^*IL-2*^ strain with *Foxp3*^*Cre*^ mice (Rubtsov et al., 2008) to create a system in which the silencing of *Il2* in Tregs was overridden (Fig. 4 A). A dose-dependent effect on splenic cellularity and mouse mortality (Fig. 4, B and C) was observed, with *Foxp3*^*Cre/wt*^ female mice (in which only 50% of Tregs would activate *Rosa*^*IL-2*^, due to X-chromosome inactivation) remaining healthy until nearly a year of age, while *Foxp3*^*Cre*^ male mice (with 100% Treg activation of *Rosa*^*IL-2*^) developed lymphoproliferation and required culling at ∼5 mo of age. Immunological assessment identified largely stable leukocyte composition (Fig. 4, D and E; and Fig. S2). In line with the effect on mortality, it was only in the *Foxp3*^*Cre*^ *Rosa*^*IL-2*^ mice that CD8 T cell numbers rose and CD4 T cell numbers collapsed (Fig. 4 F). Conversely, Tregs showed a graduated response in numbers (Fig. 4, F and G), while remaining phenotypically similar (Fig. 4 H). These systemwide data are consistent with prior work demonstrating priority access of Tregs to IL-2 and identify a threshold of cellular IL-2 provision at which CD8 T cells can become major IL-2 responders.

**Figure 5.**
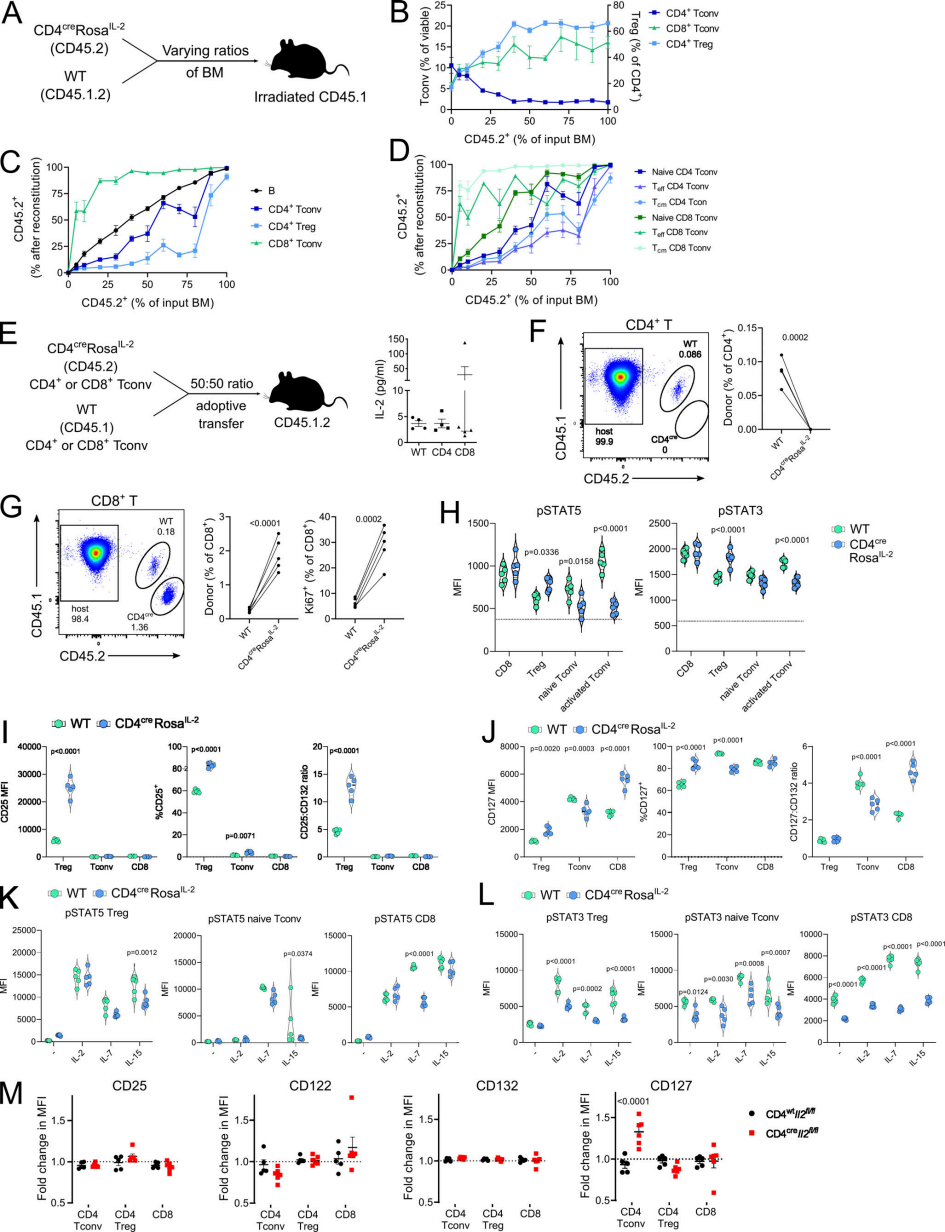
**Autocrine IL-2 production drives divergent responses in CD4**^**+**^ **and CD8**^**+**^ **T cells.** Chimeric mice were generated using BM from WT (CD45.1^+^) and CD4^cre^*Rosa*^*IL-2*^ (CD45.2^+^) at varying ratios. *n* = 4–6, pooled from two independent experiments. **(A)** Schematic of experimental outline. **(B)** Frequency of CD4^+^ and CD8^+^ Tconv cells (as percentage of viable splenocytes) or CD4^+^ Tregs (as percentage of total CD4^+^ T cells). **(C)** Frequency of splenocyte population derived from CD4^cre^*Rosa*^*IL-2*^ (CD45.2^+^) BM after reconstitution versus frequency of input BM. **(D)** Frequency of naive (CD44^lo^CD62L^hi^), Teff (CD44^hi^CD62L^lo^), or Tcm (CD44^hi^CD62L^hi^) cells derived from CD4^cre^*Rosa*^*IL-2*^ (CD45.2^+^) in spleen. **(E)** CD4^+^CD25^−^ or CD8^+^ T cells from WT and CD4^cre^Rosa^IL-2^ mice were adoptively transferred at equal ratios into immunocompetent congenic mice. Serum IL-2 levels are shown for control mice and mice that received CD4 or CD8 T cells (*n* = 4–5). **(F)** Frequency of donor cells among total CD4^+^ Tconv cells 2 wk after transfer; *n* = 4. **(G)** Frequency of donor cells among total CD8^+^ Tconv cells and Ki67 expression 2 wk after transfer; *n* = 5. **(H)** pSTAT5 and pSTAT3 in freshly isolated T cells. *F* minus one level indicated by dashed line. **(I)** Mean fluorescence intensity (MFI) and percentage of cells expressing CD25 and CD25/CD132 (common γ chain) MFI ratio on CD4^+^ Treg, CD4^+^ Tconv, and CD8^+^ T cells in WT and CD4^Cre^ Rosa^IL-2^ transgenic mice. **(J)** MFI and percentage of cells expressing CD127 (IL-7Rα) and CD127/CD132 MFI ratio on CD4^+^ Treg, CD4^+^ Tconv, and CD8^+^ T cells in WT and CD4^Cre^ Rosa^IL-2^ transgenic mice. **(K)** Upregulation of pSTAT5 in response to cytokine stimulation in CD4^+^ Treg, naive CD4^+^ Tconv, and CD8^+^ T cells. **(L)** Upregulation of pSTAT3 in response to cytokine stimulation in CD4^+^ Treg, naive CD4^+^ Tconv, and CD8^+^ T cells. **(M)** Fold-change in MFI of receptor expression in CD45.2^+^ cells to CD45.1^+^ cells in chimeric mice generated as 50% CD4^cre^*Il2*^*fl/fl*^ or CD4^wt^*Il2*^*fl/fl*^ (CD45.2^+^):50% WT (CD45.1^+^). Significance was tested by paired *t* test (F and G) or Sidak’s multiple comparison test on two-way ANOVA (H–M).

**Figure S3.**
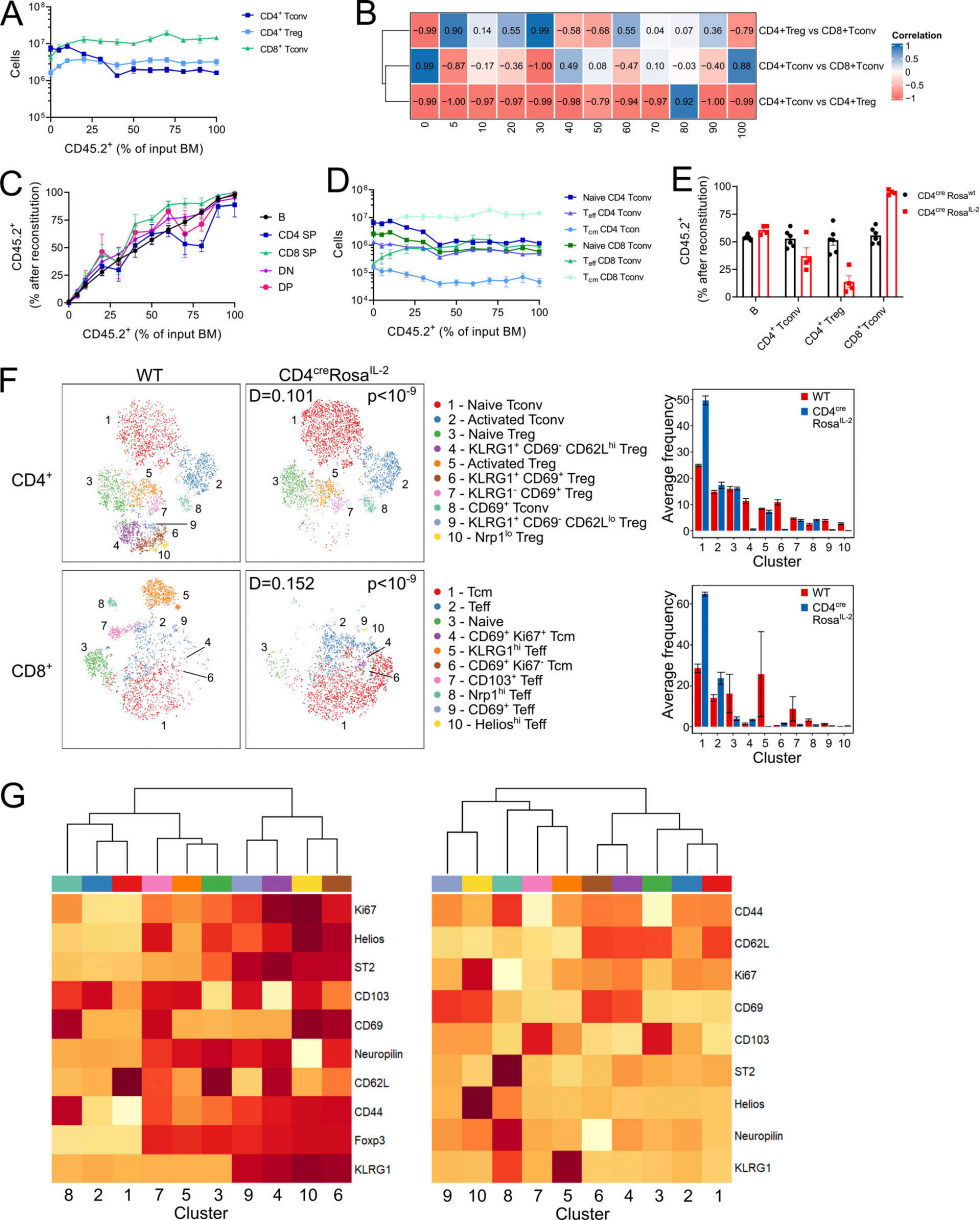
**Autocrine IL-2 production drives divergent responses in CD4**^**+**^ **and CD8**^**+**^ **T cells.** Chimeric mice were generated using BM from WT (CD45.1^+^) and CD4^cre^Rosa^IL-2^ (CD45.2^+^) at varying ratios. *n* = 4–6, pooled from two independent experiments. **(A)** Total T cell numbers in spleen. **(B)** Correlation between indicated cell types. **(C)** Frequency of thymocyte subsets derived from CD4^cre^Rosa^IL-2^ (CD45.2^+^) after reconstitution. DP, double-positive; SP, single-positive. **(D)** Total number of naive (CD44^lo^CD62L^hi^), Teff (CD44^hi^CD62L^lo^), or Tcm (CD44^hi^ CD62L^hi^) cells in the spleen. **(E)** Comparison of splenocyte frequencies from experimental chimeras (50% WT, 50% CD4^cre^Rosa^IL-2^) with control chimeras (50% WT, 50% CD4^cre^Rosa^wt^) generated simultaneously. **(F)** tSNE representation of high-parameter flow cytometry data from splenic CD4^+^ (top) and CD8^+^ (bottom) T cells from 50% WT:50% CD4^cre^*Rosa*^*IL-2*^ chimeras. FlowSOM clusters annotated based on differential expression of key markers. D value represents cross-entropy distance between samples. **(G)** Heatmap showing differential marker expression in annotated FlowSOM clusters for CD4^+^ T cells (left) or CD8^+^ T cells (right).

The titrated response of Tregs to autocrine production of IL-2 suggests that Tregs are more responsive to overall levels of IL-2 production than to autocrine sources. A limitation of the transgenic system, however, is the aggregate overproduction of IL-2. As the transgenic mouse alone cannot distinguish between effects mediated by cell-intrinsic IL-2 production and effects mediated by exposure to high serum IL-2 levels, we turned to cell transfer models to directly test the cell-intrinsic hypothesis. First, we created a titration of CD4-Cre *Rosa*^*IL-2*^ bone marrow (BM) with congenically labeled WT cells (Fig. 5 A). The system allows the simultaneous dissection of dose–response effects and autocrine/paracrine effects. The dose–response effects demonstrated a linear phase between 0–40% CD4-Cre *Rosa*^*IL-2*^ BM chimerism, where additional IL-2–producing T cells resulted in increased Treg and decreased CD4 T cells, followed by an early plateau phase at which additional IL-2 did not perturb the system further (Fig. 5 B). CD8 T cells also increased in number, with higher representation of IL-2–producing BM (Fig. 5 B; and Fig. S3, A and B), suggesting a dose-dependent response for CD8 lymphoproliferation. The use of congenic BM to titrate allowed us to determine whether there was an additional benefit of autocrine IL-2 production (i.e., a disproportionate increase in cells originating from CD4-Cre *Rosa*^*IL-2*^ BM) in addition to the dose-dependent effects. Thymic subsets of T cells remained proportional to BM chimerism, indicating that the initial differentiation process was unaffected by autocrine IL-2 (Fig. S3 C). Once in the periphery, compared with B cells as an internal control, CD8 T cells demonstrated a clear autocrine advantage when genetically licensed to produce IL-2 in an activation-independent manner (Fig. 5 C). Consistent with data from the CD8-Cre *Rosa*^*IL-2*^ mouse, the Tcm CD8 T cell subset had a preferential autocrine advantage (Figs. 5 D and S3 D). CD4 T cells, by contrast, exhibited a clear autocrine disadvantage (Fig. 5 C), demonstrating that the reduction of CD4 T cells observed in IL-2–producing strains was not due to excessive suppression by Tregs, but rather to a novel mechanism of autocrine IL-2–mediated negative feedback. Surprisingly, while Treg numbers expanded in response to more IL-2 production (Fig. 5 B), not only was no autocrine advantage observed, but a strong autocrine disadvantage was displayed (Fig. 5 C). This autocrine disadvantage was disproportionately observed among Tregs with a tissue-resident phenotype (Fig. S3, F and G). As an independent approach to verify the autocrine effect, we turned to adoptive transfer (Fig. 5 E). Adoptive transfer of equal numbers of WT and CD4-Cre *Rosa*^*IL-2*^ CD4 Tconv cells into congenic hosts also demonstrated the strong disadvantage of autocrine IL-2 production by CD4 T cells, with no *Rosa*^*IL-2*^ cells recovered 2 wk after transfer (Fig. 5 F), whereas transferred *Rosa*^*IL-2*^ CD8 T cells outcompeted WT CD8 T cells and showed a strong proliferative advantage (Fig. 5 G). In both transfer systems, the majority of recipient mice exhibited normal serum levels of IL-2 (Fig. 5 E), and the control cell transfer allowed the distinction between cell-intrinsic effects and environmental-exposure effects. Together, the BM chimera and adoptive transfer experiments validate the key phenotypes observed as cell-intrinsic results of IL-2 production rather than due to excessive serum IL-2 exposure alone.

To identify the source of the competitive fitness disadvantage exhibited by IL-2–producing CD4 T cells, in the transgenic, chimeric, and adoptive transfer contexts, we investigated cytokine responsiveness in CD4-Cre *Rosa*^*IL-2*^ mice. The net effect in vivo was a decrease in STAT3 Y705 and STAT5 Y694 phosphorylation in IL-2–producing CD4 Tconv cells, whereas cytokine signaling capacity was maintained or increased in IL-2–producing Tregs and CD8 T cells (Fig 5 H). Compared with WT mice, CD25 was elevated in Tregs and, to a lesser extent, CD4 Tconv cells in CD4-Cre *Rosa*^*IL-2*^ mice (Fig. 5 I). By contrast, expression of CD127 (IL-7Rα) was impaired in IL-2–producing CD4 Tconv cells but elevated in IL-2–producing CD8 T cells (Fig. 5 J). In vitro cytokine stimulation experiments indicated a pan–T cell defect in responding to IL-7 or IL-15 in IL-2–producing Treg, CD4 Tconv, or CD8 Tconv cells (Fig. 5, K and L). In vivo, we generated 50%:50% mixed BM chimeras of WT and CD4-Cre *Il2*^*flox*^ mice, allowing the comparison of IL-2–competent and –incompetent T cells in the same physiological environment. Although IL-2 receptor expression was intact, the loss of IL-2 production allowed IL-7Rα expression to increase in CD4 Tconv cells (Fig. 5 M). This reversal of phenotype in CD4 T cells with loss of expression of IL-2 demonstrates the phenotypic effect of physiological IL-2 production. Together, these results suggest that IL-2 production by T cells comes at a cost of loss of sensitivity to IL-7. With the net impact of elevated IL-2 signaling and reduced IL-7 signaling having divergent outcomes in CD4 and CD8 T cells, autocrine IL-2 production preferentially expands CD8 T cells while contracting CD4 T cells.

### Context-dependent perturbation of IL-2 production reveals novel cellular circuits

**Figure 6.**
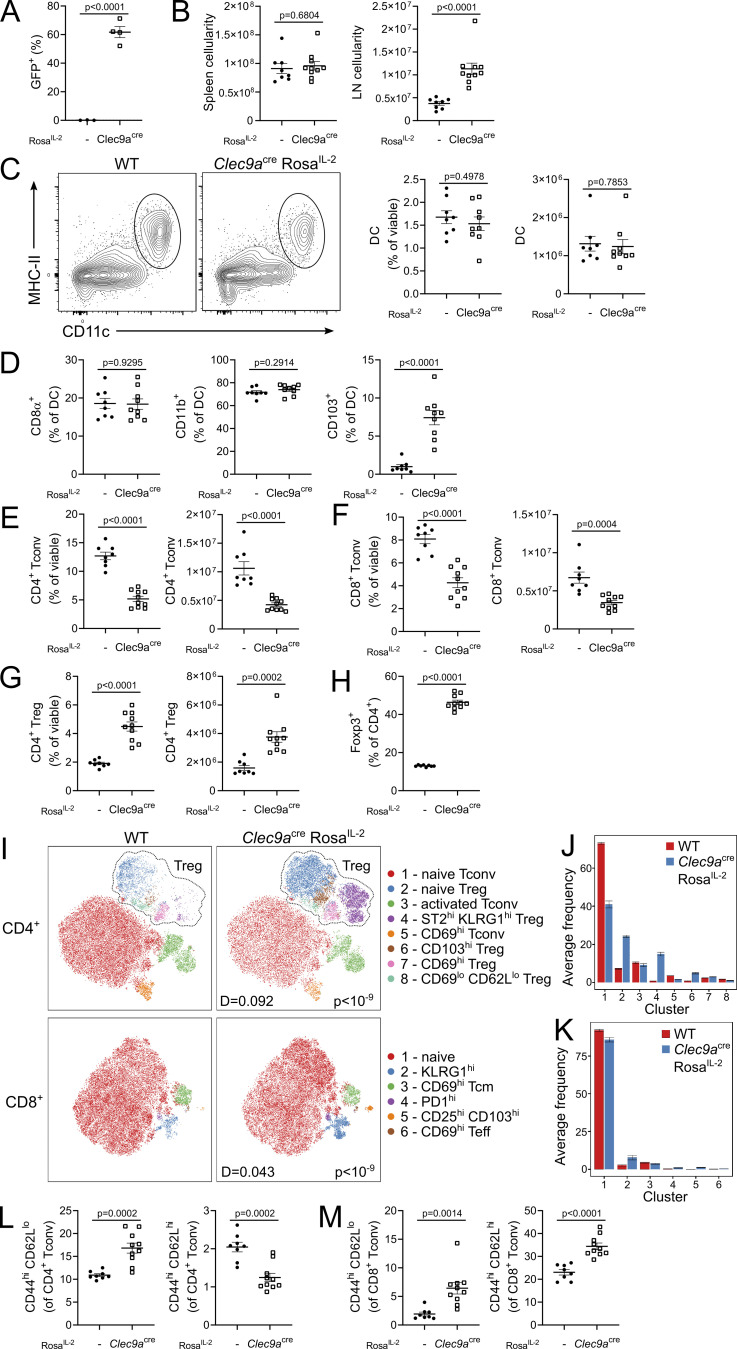
**DC-driven IL-2 favors Treg expansion. (A)** Frequency of GFP expression among DC subsets. **(B)** Cellularity of spleen and LN of 4–6-wk-old *Clec9a*^cre^ *Rosa*^*IL-2*^ and littermate controls. **(C)** Representative gating, frequency, and number of splenic DCs. **(D)** Frequency of CD8a^+^, CD11b^+^, and CD103^+^ splenic DCs. **(E–G)** Frequency and number of CD4^+^ Tconv (E), CD8^+^ Tconv (F), and CD4^+^ Treg (G) in spleen. **(H)** Frequency of Foxp3^+^ cells among total CD4 T cells. **(I)** Representative tSNE of high-parameter flow cytometry data from splenic CD4^+^ (top) and CD8^+^ (bottom). **(J and K)** Average frequency of each cluster per mouse for CD4 T cells (J) or CD8 T cells (K). **(L and M)** Frequency of effector (CD44^hi^ CD62L^lo^) or central memory (CD44^hi^ CD62L^hi^) cells from CD4 Tconv (L) or CD8 Tconv (M) cells. Data pooled from three independent experiments with 8–10 mice per genotype. Significance was tested by unpaired *t* test (A–H, L, and M) or two-sample Kolmogorov–Smirnov test (I).

**Figure 7.**
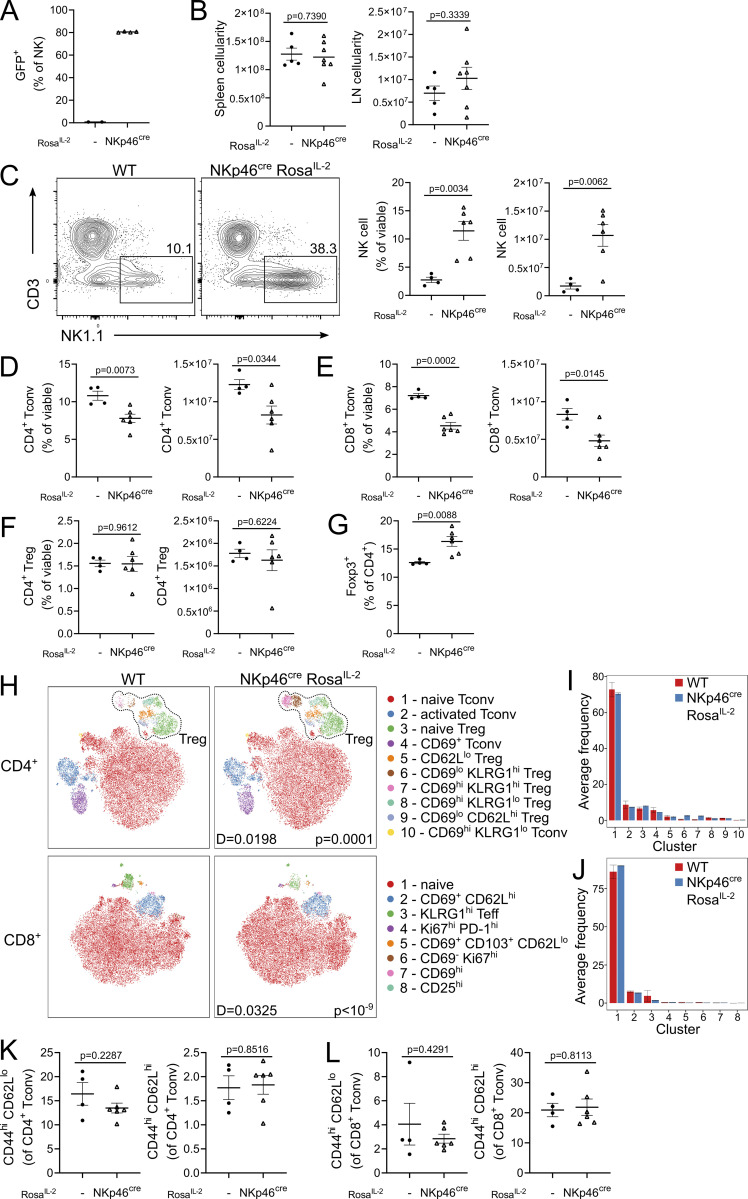
**NK cell–driven IL-2 selectively favors NK expansion. (A)** Frequency of GFP expression among NK cells. **(B)** Cellularity of spleen and LN of 4–6-wk-old NKp46^cre^ *Rosa*^*IL-2*^ and littermate controls. **(C)** Representative gating, frequency, and number of splenic NK cells. **(D–F)** Frequency and number of CD4 Tconv (D), CD8 Tconv (E), and CD4 Treg (F) cells in spleen. **(G)** Frequency of Foxp3^+^ cells among total CD4^+^ T cells. **(H)** Representative tSNE of high-parameter flow cytometry data from splenic CD4^+^ (top) and CD8^+^ (bottom). **(I and J)** Average frequency of each cluster per mouse for CD4 T cells (I) or CD8 T cells (J). **(K and L)** Frequency of effector (CD44^hi^CD62L^lo^) or central memory (CD44^hi^CD62L^hi^) cells from CD4 Tconv (K) or CD8 Tconv (L) cells. Data pooled from two independent experiments with four to six mice per genotype. Significance was tested by unpaired *t* test (A–G, K, and L) or two-sample Kolmogorov–Smirnov test (H).

To test for contextual symmetry in the IL-2 network, we amplified three existing but minor IL-2 sources, DCs, NK cells, and B cells, through crossing the *Rosa*^*IL-2*^ allele onto the *Clec9a*^*Cre*^, *Ncr1*^*Cre*^ (NKp46-Cre), and *Cd19*^*Cre*^ strains, respectively. *Clec9a*^*Cre*^ *Rosa*^*IL-2*^ mice, with basal production of IL-2 from DCs (Fig. 6 A), displayed an increase in cellularity of the LN but not spleen (Fig. 6 B). These mice maintained a normal number of DCs, with the exception of a large increase in the CD103^+^ population (Fig. 6, C and D). Responding populations were largely restricted to Tregs, in particular those with a resident-like phenotype (Fig. 6, E–K). Unlike the observations using T cell drivers of IL-2, CD8 T cells were reduced rather than expanded (Fig. 6 F), with a more pronounced loss of cells with a Tcm phenotype (Fig. 6, L and M). Previous studies have demonstrated a contribution of DC-derived IL-2 to Treg homeostasis, with DC-derived IL-2 able to partially sustain LN Treg numbers in the absence of T cell–derived IL-2 (Owen et al., 2018). In combination with our data, this suggests a primary wiring of DC-derived IL-2 to the Treg sink. NKp46-Cre *Rosa*^*IL-2*^ mice, with basal production of IL-2 from NK cells and ILC3s, displayed relatively unaltered gross cellularity (Fig. 7, A and B). However, there was a large (∼10-fold) and specific expansion of the NK population, with unaltered Treg numbers and a small decline in CD4 and CD8 Tconv numbers (Fig. 7, C–L). These data suggest that, like CD8 T cells, NK cells preferentially respond to intrinsic production of IL-2.

**Figure 8.**
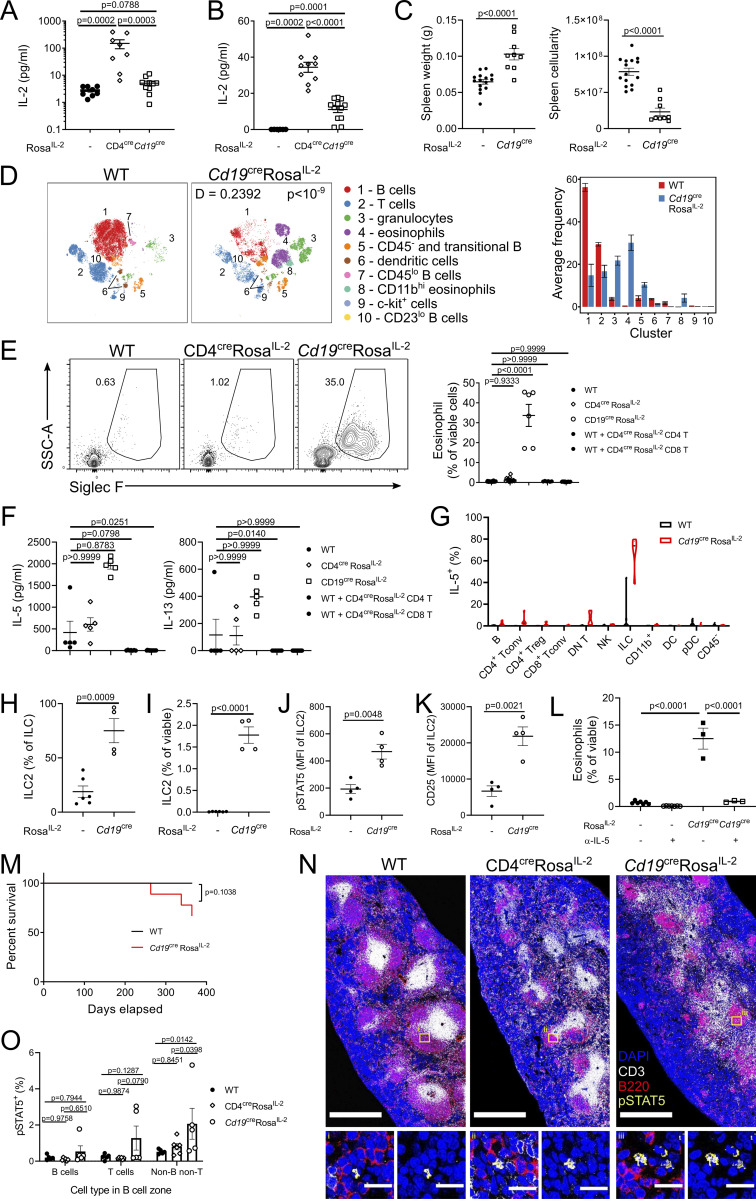
**Expression of IL-2 by B cells drives a distinct ILC2-eosinophil–oriented cellular circuit. (A)** ELISA of IL-2 cytokine in serum. *n* = 8–10. **(B)** ELISA of IL-2 expression in splenic tissue. *n* = 6–12. **(C)** Spleen weight and cellularity of 4–6-wk-old *Cd19*^cre^ *Rosa*^*IL-2*^ and littermate controls. *n* = 9–15. **(D)** Representative tSNE of high-parameter flow cytometry data from splenic viable cells and average frequency of each cluster per mouse. **(E)** Representative gating and frequency of eosinophils in spleen from WT, CD4^cre^Rosa^IL-2^, *Cd19*^cre^Rosa^IL-2^, or WT mice transferred with CD4^cre^Rosa^IL-2^ CD4^+^ or CD8^+^ T cells. *n* = 6–25. SSC, side scatter. **(F)** Luminex analysis of indicated cytokine in serum. *n* = 5–15. **(G)** IL-5 expression among cell lineages in spleen. *n* = 6–9. pDC, plasmacytoid DC. **(H)** Frequency of GATA3^+^ ILC2 among total ILCs in spleen. *n* = 4–6. **(I)** Frequency of ILC2 in spleen. *n* = 4–6. **(J)** pSTAT5 in freshly isolated ILC2 cells. *n* = 4. MFI, mean fluorescence intensity. **(K)** CD25 MFI of ILC2 in spleen. *n* = 4. **(L)** Frequency of splenic eosinophils in mice treated with anti−IL-5 neutralizing antibody. *n* = 3–8. **(M)** Survival analysis. *n* = 7–9. **(N)** Representative immunofluorescence staining of pSTAT5, CD3, and B220 in spleen (scale bars: 500 μm; 15 μm for inset). *n* = 5–6. **(O)** Frequency of pSTAT5^+^ cells found in splenic B cell zones by immunofluorescence. *n* = 5–6. Data pooled from (A–E and G–O) or representative of (F) at least two independent experiments. Significance was tested by one-way ANOVA (A, B, E, F, and L), unpaired *t* test (C and H–K), two-sample Kolmogorov–Smirnov test (D), Mantel–Cox log-rank test (M), or two-way ANOVA (O).

**Figure S4.**
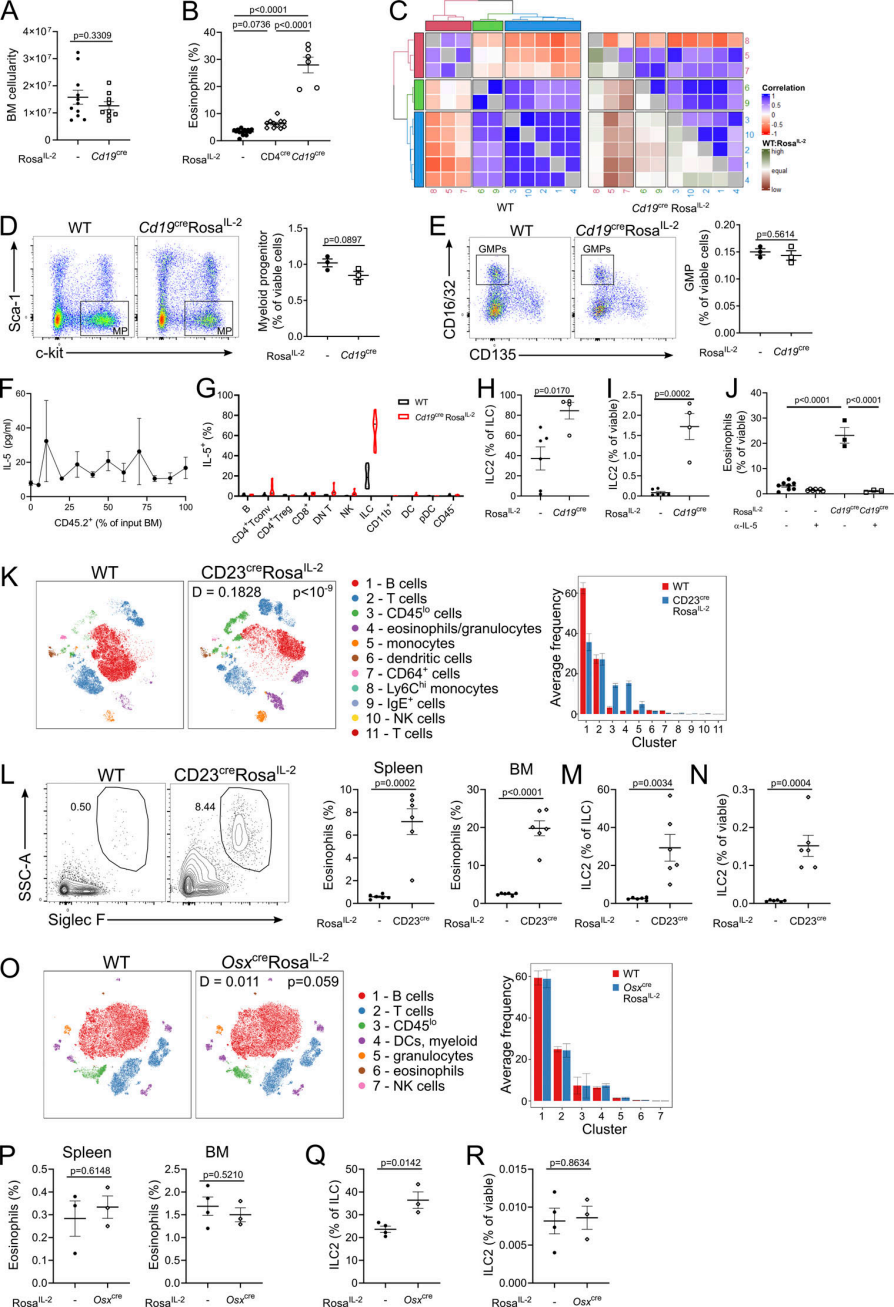
**Expression of IL-2 by mature B cells, but not BM-localized IL-2, drives the ILC2-eosinophil circuit. (A)** BM cellularity of 4–6-wk-old *Cd19*^*cre*^ *Rosa*^*IL-2*^ mice and littermate controls. *n* = 9–11. **(B)** Frequency of eosinophils in the BM. *n* = 6–14. **(C)** Correlation between clusters in Fig. 8 D. WT:Rosa^IL-2^ indicates whether correlation is higher or lower in WT relative to *Cd19*^cre^ Rosa^IL-2^. **(D)** Representative gating and frequency of myeloid progenitors (MP; lineage^−^Sca-1^−^c-kit^+^) cells in the BM, pregated on CD45^+^lineage^−^. *n* = 3. **(E)** Representative gating and frequency of GMPs (granulocyte-monocyte progenitors) in the BM (pregated from MP gate). *n* = 3. **(F)** Serum IL-5 in mixed WT/CD4^cre^Rosa^IL-2^ BM chimeras as generated in Fig. 5 A. **(G)** IL-5 expression among cellular lineages in the BM. *n* = 6–9. pDC, plasmacytoid DC. **(H)** Frequency of GATA3^+^ ILC2 among total ILCs in BM. *n* = 4–6. **(I)** Frequency of ILC2 in BM. *n* = 4–6. **(J)** Frequency of eosinophils in the BM of mice treated with anti−IL-5 neutralizing antibody. *n* = 3–8 mice. **(K)** Representative tSNE of high-parameter flow cytometry data from 4–6-wk-old *CD23*^*cre*^ *Rosa*^*IL-2*^ mice and littermate control splenic viable cells and average frequency of each cluster per mouse. **(L)** Representative gating and frequency of eosinophils in spleen and BM. *n* = 6. SSC, side scatter. **(M)** Frequency of GATA3^+^ ILC2 among total ILCs in spleen. **(N)** Frequency of ILC2 in spleen. **(O)** Representative tSNE of high-parameter flow cytometry data from 4–6-wk-old *Osx*^cre^ *Rosa*^*IL-2*^ and control splenic viable cells and average frequency of each cluster per mouse. **(P)** Frequency of eosinophils in spleen and BM. *n* = 3. **(Q)** Frequency of GATA3^+^ ILC2 among total ILCs in spleen. **(R)** Frequency of ILC2 in spleen. Significance was tested by unpaired *t* test (A, D, E, H, I, L–N, and P–R), one-way ANOVA (B and J), or two-sample Kolmogorov–Smirnov test (K and O).

Surprisingly, production of IL-2 from B cells, using *Cd19*^*Cre*^ *Rosa*^*IL-2*^ mice, drove a distinct cellular network to all other IL-2 sources, despite levels of net IL-2 production in serum (Fig. 8 A) and splenic tissue (Fig. 8 B) elevated to a lesser degree than observed in CD4^Cre^ *Rosa*^*IL-2*^ mice. *Cd19*^*Cre*^ *Rosa*^*IL-2*^ mice exhibited an enlarged spleen, but with reduced splenic cellularity due to extensive fibrosis (Fig. 8 C). Assessment of leukocyte changes identified the primary shift as a 50-fold increase in eosinophils, which was not observed in CD4^cre^Rosa^IL-2^ mice or in WT mice transferred with CD4^cre^Rosa^IL-2^ CD4 or CD8 T cells (Fig. 8, D and E; and Fig. S4). Eosinophilia was accompanied by large increases in IL-13 and, especially, IL-5 (Fig. 8 F). ILCs were the dominant source of IL-5 in *Cd19*^*Cre*^ *Rosa*^*IL-2*^ mice (Fig. 8 G), with ILC2 numbers expanded 100-fold, to constitute >90% of the ILC pool (Fig. 8, H and I). ILC2 from *Cd19*^*Cre*^ *Rosa*^*IL-2*^ mice had increased pSTAT5 Y694 phosphorylation and upregulation of CD25, indicating increased responsiveness to IL-2 (Fig. 8, J and K). A similar increase in ILC2 and eosinophils was observed in the BM (Fig. S4). To test the possibility that this phenotype is driven by early BM expression of IL-2, rather than B cell–specific expression, we made the additional crosses to *CD23*^*Cre*^ and *Osx*^*Cre*^ mice. *CD23*^*Cre*^, despite turning on later in mature B cells, generated the same eosinophil-dominated phenotype (Fig. S4, K–N), while *Osx*^*Cre*^, active in BM osteoblasts, did not (Fig. S4, O–R). Neutralization of IL-5 in *Cd19*^*Cre*^ *Rosa*^*IL-2*^ mice prevented the eosinophilia (Figs. 8 L and S4 J), demonstrating that B cell–specific production of IL-2 initiated an unconventional cellular circuit expanding IL-5–expressing ILC2s and, downstream, eosinophils. This immune dysregulation did not result in significant excess mortality (Fig. 8 M), unlike that observed with the T cell Cre drivers (Figs. 2 K and 4 C). Histological assessment of the spleen found altered composition of the white pulp in *Cd19*^*Cre*^ *Rosa*^*IL-2*^ mice, with T cell zones reduced in both size and T cell density, compared with WT or CD4^Cre^ *Rosa*^*IL-2*^ mice (Fig. 8 N), consistent with the reduced frequency of T cells observed in these mice by flow cytometry (Fig. 8 D). Within the B cell zones, relative to both WT or CD4^Cre^ *Rosa*^*IL-2*^ mice, *Cd19*^*Cre*^ *Rosa*^*IL-2*^ mice demonstrated elevated rates of pSTAT5 in non-T, non-B cells (Fig. 8 O). Together, these data suggest that physical proximity to B cells provides certain lymphocytes access to IL-2 when sourced from B cells, triggering the ILC2-eosinophil cascade.

**Figure 9.**
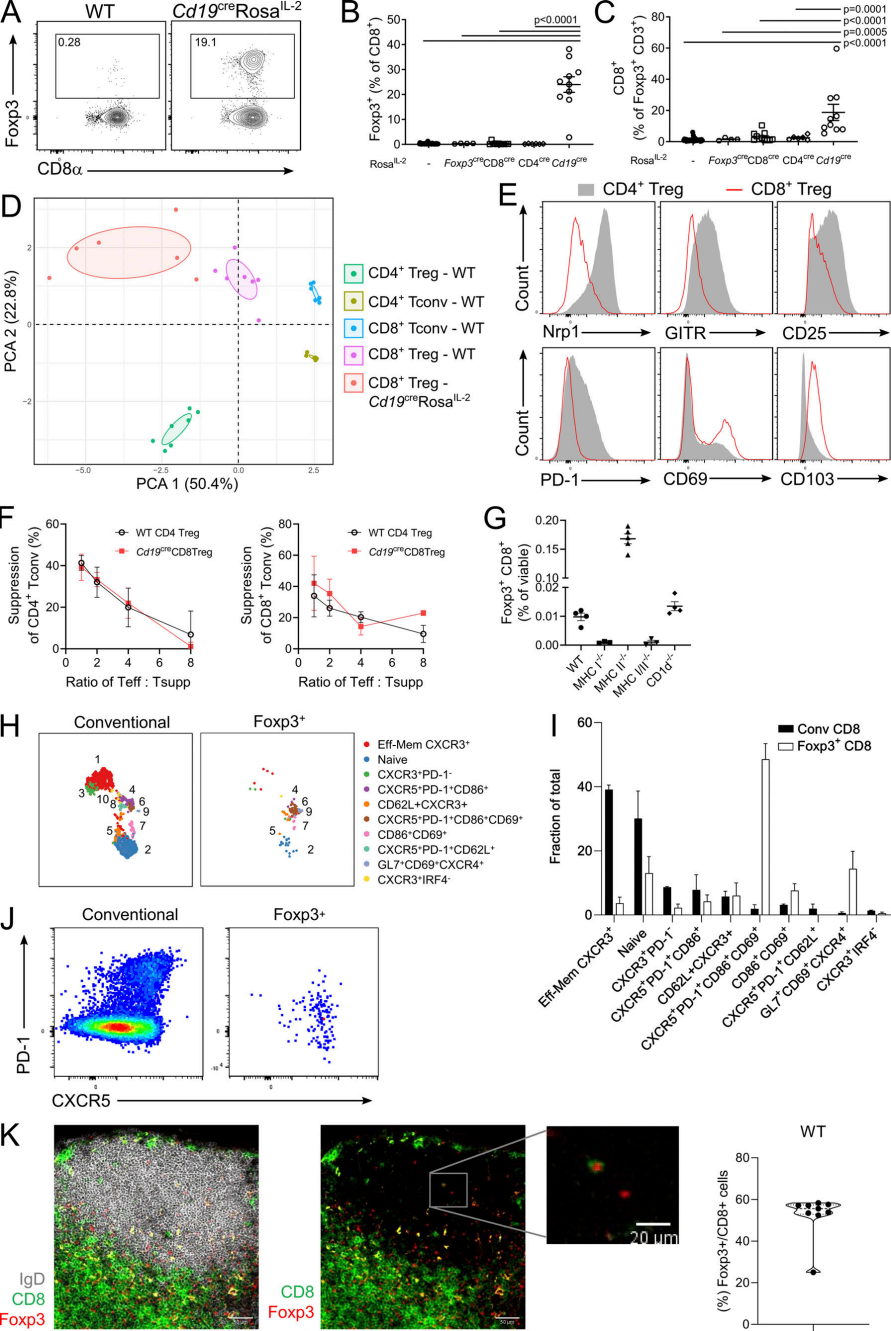
**CD8**^**+**^**Foxp3**^**+**^ **Tregs are revealed through B cell production of IL-2. (A)** Representative gating of Foxp3^+^ CD8^+^ T cells in *Cd19*^cre^Rosa^IL-2^ mice and littermate controls. **(B)** Frequency of Foxp3 expression among CD8^+^ T cells in Rosa^IL-2^ strains. *n* = 6–32. **(C)** Frequency of CD8^+^ cells among total Foxp3^+^ T cells. *n* = 9–32. **(D)** Principal component analysis of flow cytometric markers on splenic T cell populations. **(E)** Representative expression of indicated markers on CD4^+^ or CD8^+^ Tregs from *Cd19*^cre^Rosa^IL-2^ mice. **(F)** In vitro suppression assay comparing ability of CD4^+^ and CD8^+^ Tregs to suppress CD4^+^ Tconv (left) or CD8^+^ T conv (right) cell proliferation; pooled from three independent experiments. **(G)** Frequency of Foxp3^+^CD8^+^ cells in spleens of WT, *MHCI*^*−/−*^, *MHCII*^*−/−*^, *MHCI*^*−/−*^*MHCII*^*−/−*^, and *CD1d*^*−/−*^ mice. **(H and I)** UMAP of high-parameter flow cytometry data (H) from Foxp3^−^CD8^+^ Tconv cells and CD8^+^ Tregs, with frequency distribution (I) of Foxp3^+^ and CD8 Tconv cells across FlowSOM clusters (*n* = 3). **(J)** Representative flow plot showing PD-1 and CXCR5 expression. **(K)** Representative immunofluorescence staining of CD8α, Foxp3, and IgD of WT LN (scale bars: 50 μm; 20 μm for inset), with quantification of Foxp3^+^ cells among total CD8 T cells in the B follicle (*n* = 9). Data pooled from (B–D and F–I) or representative of (A, E, J, and K) at least two independent experiments. Significance was tested by one-way ANOVA (B, C, and G).

**Figure S5.**
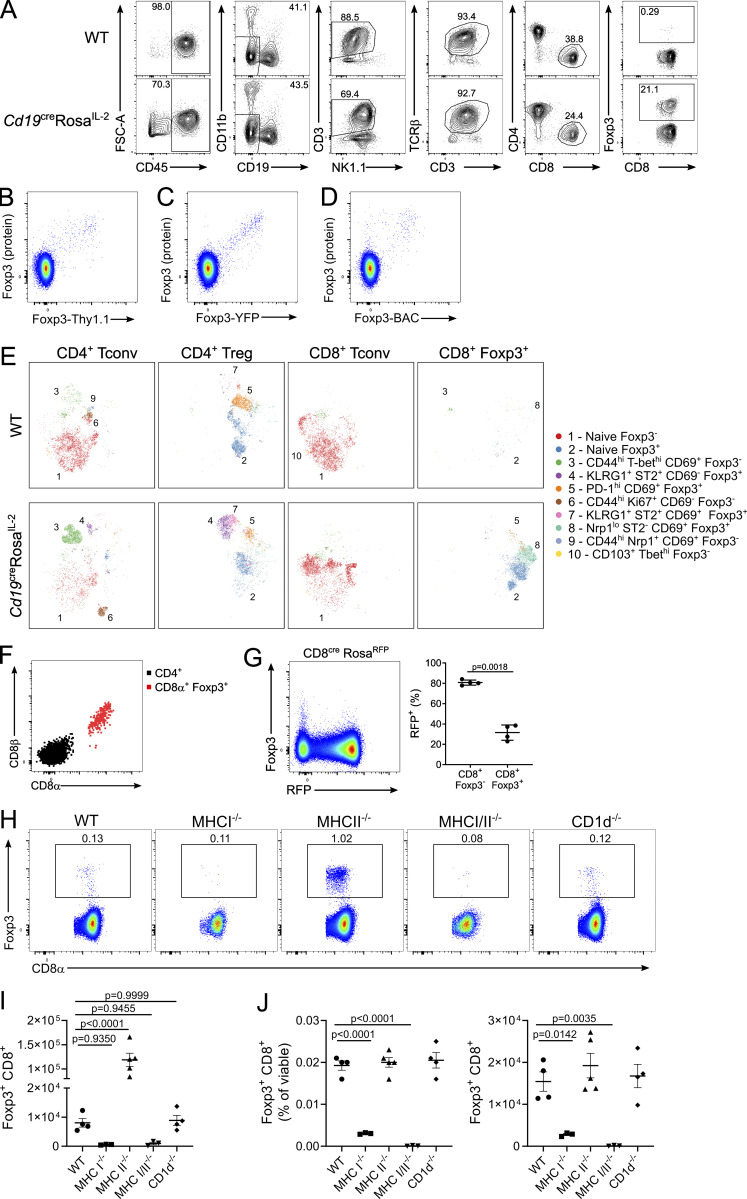
**Foxp3 expression in the CD8 lineage. (A)** Hierarchical gating of CD8α^+^Foxp3^+^ cells in *Cd19*^cre^Rosa^IL-2^ and littermate controls, pregated on viable lymphocytes. FSC, forward scatter. **(B–D)** Expression of the *Foxp3*^Thy1.1^ reporter (B), *Foxp3*^YFP-cre^ reporter (C), and Foxp3^BAC^ reporter (D) in CD8α^+^CD4^−^ T cells. **(E)** tSNE representation of high-parameter flow cytometry data from splenic T cell populations from *Cd19*^cre^Rosa^IL-2^ mice and littermate controls. FlowSOM clusters annotated based on differential expression of key markers. **(F)** Expression of CD8β in CD8α^+^Foxp3^+^ cells from the spleen of WT mice. **(G)** Usage of E8I enhancer (RFP^+^) in CD8^+^ T cells from CD8^cre^Rosa^RFP^ mice. *n* = 4, unpaired *t* test. **(H)** Representative gating of CD8^+^Foxp3^+^ cells in spleens of WT, *MHCI*^*−/−*^, *MHCII*^*−/−*^, *MHCI*^*−/−*^ *MHCII*^*−/−*^, and *CD1d*^*−/−*^ mice. **(I)** Number of CD8^+^Foxp3^+^ cells in spleen. **(J)** Frequency and number of CD8^+^Foxp3^+^ cells in thymus. For H–J, *n* = 3–5, data pooled from two independent experiments. Significance tested by one-way ANOVA.

Finally, we observed a second major abnormality in *Cd19*^*Cre*^ *Rosa*^*IL-2*^ mice: a 40-fold expansion of CD8^+^Foxp3^+^ cells (Fig. 9, A–C). This population, exceedingly rare in WT mice, expanded to ∼20% of CD8 T cells in *Cd19*^*Cre*^ *Rosa*^*IL-2*^ mice. Foxp3 expression in CD8^+^ Foxp3^+^ cells correlated with three independent Foxp3 marker systems (Fig. S5, A–D). Notably, the provision of IL-2 to drive this increase needed to come from B cells, as the population remained small in mice with the CD4-Cre or CD8-Cre drivers (Fig. 9, B and C). CD8^+^ Foxp3^+^ cells displayed a distinct phenotype from either CD8 Tconv cells or CD4 Tregs (Figs. 9 D and S5 E), with expression of some Treg markers, such as CD25, but low expression of others, such as Nrp1 (Fig. 9 E). The CD8^+^Foxp3^+^ cells expanded in *Cd19*^*Cre*^ *Rosa*^*IL-2*^ mice were, however, bona fide Tregs, with an in vitro suppressive capacity on both CD4 and CD8 Tconv cells indistinguishable from that of CD4^+^Foxp3^+^ cells (Fig. 9 F). Analysis of the small CD8^+^Foxp3^+^ population present in WT mice demonstrated that they were CD8αβ cells; however (and unusually), the CD8-Cre transgene had poor penetrance in the CD8^+^Foxp3^+^ population (Fig. S5, F and G). As this was akin to CD4^+^CD8^+^ double-positive thymocytes, we sought to determine whether this population were CD8^+^ MHCII-restricted cells. Comparison of WT, CD1d KO, MHCI KO, MHCII KO, and MHC I/II double KO mice demonstrated that CD8^+^Foxp3^+^ are classic MHCI-restricted CD8 T cells (Fig. 9 G and Fig. S5, H–J). High-dimensional flow cytometry profiling of CD8^+^Foxp3^+^ cells in WT mice found a highly skewed subset distribution compared with CD8^+^Foxp3^−^ Tconv cells (Fig. 9 H). In particular, splenic CD8^+^Foxp3^+^ cells were greatly enriched (∼50%) for the CXCR5^+^PD1^+^ subset (Fig. 9, I and J). As this phenotype is shared with follicular helper CD4 T cells (Vinuesa et al., 2016), we investigated the anatomic distribution of CD8^+^Foxp3^+^ cells in the spleen and found that while <1% of CD8 T cells in total, this population constituted ∼60% of CD8 T cells present in the B cell zone of naive mice (Fig. 9 K). These data provide a plausible route for the specific expansion of CD8 Tregs in *Cd19*^*Cre*^ *Rosa*^*IL-2*^ mice: their presence in the B cell zone puts them in close proximity with B cell–derived IL-2 production, while making them relatively refractory to T cell–derived IL-2. These mice, in addition to providing for sizeable populations of CD8 Tregs amenable to functional assays, therefore illustrate the contextual importance of IL-2 production.

## Discussion

Here we developed a genetic tool allowing for the directed production of IL-2 production. The levels of production achieved, while lower than those of endogenous IL-2 on a per-cell basis, are elevated above the physiological range present in unmanipulated mice, owing to the expanded cellular sources and the constitutive nature of the production. The level of overexpression observed here (∼100 pg/ml in the serum of CD4-Cre *Rosa*^*IL-2*^ mice and ∼5 pg/ml in the serum of *Cd19*^*Cre*^ *Rosa*^*IL-2*^ mice) are in the range of those observed in mice with genetic deletion of CD25 (Sharma et al., 2007) and in line with the IL-2 overproduction observed in certain human inflammatory diseases, such as tuberculous (∼160 pg/ml; Berktas et al., 2004), rheumatoid arthritis (∼240 pg/ml; Oncul et al., 2002), celiac disease (∼60 pg/ml; Goel et al., 2020), or myelofibrosis (∼970 pg/ml; Panteli et al., 2005). Elevated IL-2 levels are also explicitly desired therapeutically in numerous disease contexts, and patients treated with therapeutic IL-2 exhibit even higher serum IL-2 levels, with studies showing >3,000 pg/ml after high-dose IL-2 therapy (Panelli et al., 2004) and ∼900 pg/ml after low-dose IL-2 therapy (Nguyen et al., 2019). It is therefore important to model the effects of IL-2 in concentrations above the physiologically healthy range. Although our transgenic system does not reproduce the complex context of specific diseases, the directed perturbation recapitulates phenotypes observed in IL-2–treated patients. The system therefore aids in understanding the consequences of cell type– and anatomy-restricted IL-2 production pathways invoked during certain diseases or mimicked by IL-2 treatment, permitting a broader understanding of the IL-2 network as a diverse array of potential producers and responders.

Under the standard “immunology as a single-cell suspension” perspective, IL-2 generated by any source enters a common pool, with response and consumption driven by affinity-based cellular capture. The dominant components of the IL-2 network at homeostasis are compatible with this simplified model. Conventional CD4 T cells are the dominant source, and the two main sinks are Tregs and CD8 T cells, competing for IL-2 access and responding to the signal produced (Feinerman et al., 2010). Priority within the sink populations is consistent with the affinity of receptor expression, with Tregs responding at a lower dose (and expressing the high-affinity trimeric receptor) and CD8 T cells responding at higher doses (consistent with expression of the intermediate-affinity dimeric receptor). Amplification of minor sources of IL-2, however, demonstrates the limitations inherent to the context-independent model. NK cells, ILC2s (Spolski et al., 2018), and CD8 Tregs (current study) can all express the high-affinity trimeric receptor and yet do not respond to T cell–derived IL-2. Instead, NK cells responded only to autocrine production, while ILC2s and CD8 Tregs were expanded only by B cell–sourced IL-2. This demonstrates that the range of responses possible to IL-2 is not only due to receptor expression and preferential capture based on affinity, but rather there is a context-dependent component.

While we do not negate the utility of the affinity-based competition model, these data do necessitate the inclusion of context sensitivity into the model, where the cellular source of IL-2 dictates its function. In the absence of evidence for more exotic hypotheses, such as posttranscriptional modification by the cellular source and binding to undiscovered alternative receptors (as commonly occurs for chemokines, altering receptor preference; Stone et al., 2017), the parsimonious explanation lies in a proximity-based effect. The diffusion radius of IL-2 is as low as 30 μm, with high densities of consuming cells (Oyler-Yaniv et al., 2017), which would be expected to result in a sharp IL-2 gradient at the border of the T cell zone. Indeed, Tregs responding to IL-2 are shown to colocalize within 100 μm of IL-2–producing cells (Liu et al., 2015), demonstrating the tightly restricted anatomic space in which IL-2 travels. The expansion of ILC2s and CD8 Tregs supports anatomic proximity as the contextual source, since we find CD8 Tregs heavily enriched in the B cell zone, while ILC2 cells reside in the B cell–adjacent interfollicular area (Mackley et al., 2015), consistent with their function in promoting early antibody responses (Drake et al., 2016). Proximity-based contextualism would likely result in different IL-2 circuitry in nonlymphoid organs, such as the ILC3-to-Treg link proposed in the gut (Zhou et al., 2019). IL-2 may itself contribute to rewiring of the circuit in such contexts, as IL-2 response genes are highly enriched for microenvironmental sensors (Rollings et al., 2018; Ross et al., 2016).

The relationship connecting CD4 T cell IL-2 production to Tregs and CD8 T cells is the best-described aspect of the IL-2 network. In the thymus, production of IL-2, primarily from self-reactive thymocytes (Hemmers et al., 2019; Owen et al., 2018), helps drive Treg differentiation via signaling to the CNS2 Foxp3 genetic element (Feng et al., 2014). Intriguingly, a potential role for autocrine IL-2 in the early thymic Treg precursor has been identified (Chawla et al., 2020). In the periphery, IL-2 production from activated CD4 T cells is critical to support the fitness (Fontenot et al., 2005), survival (Pierson et al., 2013; Shi et al., 2018), and regulatory function (Chinen et al., 2016) of Tregs. CD8 T cells are highly dependent on IL-2 for the formation of memory (Williams et al., 2006), setting up a competitive dynamic between Tregs and CD8 T cells for IL-2 consumption (Pandiyan et al., 2007). Indeed, the preferential ability of Treg to capture IL-2, based on expression of CD25, impairs the ability of CD8 T cells to enter the memory fate (Chinen et al., 2016). IL-2 is not a passive molecule, simply consumed, but in turn drives molecular changes that alter downstream cellular competition, such as the expansion of the Treg population. These effects can be complex, as elevated Tregs not only suppress bulk CD8 T cell responses, but benefit the quality of antigen-specific CD8 T cell responses, via more complete suppression of low-affinity clones (Pace et al., 2012). The extraordinary capacity of both Tregs and CD8 T cells to respond to IL-2 dictates the highly dependent nature these cells have on CD4 Tconv cells, their primary source. Such a system provides a failsafe to prevent runaway proliferation, as observed when we permit the normally forbidden constitutive expression in these restricted lineages. Notably, however, the outsourcing of IL-2 production to CD4 Tconv cells shifts the risk of a positive feedback loop onto the source cell type. Here, the toxicity cost of IL-2 production, rather than being an inadvertent metabolic cost, may serve as an engineered regulatory check on a potential positive feedback loop. We identified in vivo the same effect previously observed in vitro of IL-2–mediated downregulation of IL-7 receptor expression (Xue et al., 2002). The ability of IL-2 to signal within the endosomal compartment (Konstantinidis et al., 2005; Volko et al., 2019) may provide the mechanistic route through which IL-2 production drives competitive costs in CD4 T cells, as the IL-7 receptor sequestration process could occur during the process of IL-2 secretion. This coupling of IL-2 production to IL-7 desensitization may thus provide a production-cost mechanism to IL-2–producing CD4 T cells. Intriguingly, the same endosomal signaling pathway could account for the competitive advantage when initiated in CD8 T cells, as the endosomal compartment concentrations may compensate for the lower affinity of the dimeric receptor. The network analysis thus reveals elegant details even for the best-described IL-2 cellular circuits.

Foxp3^+^ CD8 Tregs, distinct from other CD8 populations with proposed suppressive capacity, have been previously described in mouse and human (Mayer et al., 2011; Vieyra-Lobato et al., 2018). The CD8^+^Foxp3^+^ population is extremely low, 0.1% of CD8 T cells in mice and 0.3% in humans (Churlaud et al., 2015). The CD8^+^Foxp3^+^ population has been reported to expand in patients in response to IL-2 treatment (Rosenzwajg et al., 2015), indicating that the response to IL-2 observed here has a cross-species analog, and in the inflammatory contexts of animal models of allogeneic transplantation (Beres et al., 2012; Robb et al., 2012) or retrovirus infection (Nigam et al., 2010). The extremely low numbers have precluded ready functional validation or wide acceptance among the immunological community. The existence of a link between CD8 Tregs and B cells, identified here, provides both a novel insight into the biology of this neglected population and also a tool for study, allowing CD8 Tregs to be purified in numbers akin to CD4 Tregs. While the expression of Foxp3 marks these CD8 Tregs as a distinct lineage from various Foxp3^−^ CD8 lineages with regulatory functions (Niederlova et al., 2021), it is a notable convergence that several of these Foxp3^−^ CD8 lineages have been demonstrated to inhibit T follicular helper cells and autoantibody production (Kim et al., 2010; Mishra et al., 2021), suggesting a multilineage cooperation between CD8 regulatory lineages and control over B cell responses.

The anomalous circuit driven by B cell–derived IL-2 production provides a mechanistic explanation for a longstanding puzzle of IL-2 clinical use. As IL-2 is an ectopically provided therapeutic, it is critical to understand the physiological effects of “overexpressed” IL-2 distorting the homeostatic circuit. In the first clinical trials, in the 1980s, of recombinant human IL-2 in primary immunodeficiency (Dopfer et al., 1984), AIDS (Kern et al., 1985), and cancer (Macdonald et al., 1990b), patients almost invariably developed eosinophilia, accompanied by high IL-5 titers. Despite treatment modification to reduce toxicity, similar results are a longstanding feature of IL-2 treatment for cancer, including pediatric tumors (Roper et al., 1992), renal cell carcinoma (Clark et al., 1999; Lee et al., 2010; Moroni et al., 2000), non–small cell lung cancer (Ardizzoni et al., 1994), neuroblastoma (Pardo et al., 1996), mesothelioma (Nano et al., 1998), and melanoma (Cragun et al., 2005; Woodson et al., 2004), with eosinophilia in treated patients being predictive of treatment failure (Moroni et al., 2000). Human eosinophils were reported to express IL-2 receptors (Rand et al., 1991); however, in vitro assays suggested that eosinophilia was due to IL-5, rather than direct effects of IL-2 (Macdonald et al., 1990a). IL-5–producing T cells were initially proposed as an intermediary (Enokihara et al., 1988; Enokihara et al., 1989), before the discovery of IL-5–producing ILC2 cells. Using a mouse model of IL-2–anti-IL-2 antibody complex injection, the Bluestone group demonstrated that ILC2 cells were the primary IL-5–expressing cells arising following these injections, and that ablation of all IL-5^+^ cells prevented eosinophilia (Van Gool et al., 2014).

Our results provide a potential explanation for why endogenous IL-2 production does not drive the same eosinophilic outcome, with only B cell–sourced IL-2 precipitating the ILC2 circuit. We propose that exogenous IL-2 provision violates the default anatomic restriction of major IL-2 production to T cell zones, thus precipitating a normally quarantined reaction. This is consistent with the increased frequency of pSTAT5^+^ cells observed within the B cell zone, only in mice in which B cells are producing IL-2. Intriguingly, one of the physiological contexts in which this B cell–driven circuit is naturally amplified may be that of parasitic infection. During infection with *Heligmosomoides polygyrus*, B cell production of IL-2 is required for parasite control (Wojciechowski et al., 2009). While attributed to direct support for Th2 cells, IL-2 in this context may provide indirect support via ILC2 cells (Pelly et al., 2016). Eosinophilia in the context of *H*. *polygyrus* is superfluous for clearance (Urban et al., 1991); however, amplification of this circuit would be beneficial in other helminth infections (Klion and Nutman, 2004), demonstrating the contingent value of the B cell–ILC2–eosinophil circuit identified here.

A shift from an affinity-based competition model to a context-sensitive model creates new potentials for therapeutic delivery. The discrepancy between objective and outcome in IL-2 trials has been attributed to widespread receptor expression, with extensive research going into the design of synthetic IL-2 mutants (Spangler et al., 2015). The concept behind this approach is that by restricting IL-2 impact to only one receptor, adverse effects will be eliminated. IL-2 engineering is primarily achieved via altering binding to either CD25 or CD122. Reduced binding to CD25 (Carmenate et al., 2013) or enhanced binding to CD122 (Levin et al., 2012) promotes CD8 T cell responses, whereas reduced binding to CD122 (Peterson et al., 2018) or enhanced binding to CD25 (Rao et al., 2005; Rao et al., 2003) accentuates the Treg response. More elaborate engineering includes combining mutations (Sun et al., 2019), modifications to the common CD132 chain (Mitra et al., 2015), fusion to antibody domains (Khoryati et al., 2020; Spangler et al., 2018; Sun et al., 2019), or even de novo mimics that trigger receptor binding without homology to IL-2 (Silva et al., 2019). In each case, while the engineered properties impart receptor specificity, they neglect contextual factors and therefore cannot impart cellular specificity. It is only when coupled with cell therapy that biochemical engineering of IL-2 can impart cell specificity, such as through the generation of orthogonal IL-2 treatment coupled to transfer of cells engineered to express an orthogonal receptor (Sockolosky et al., 2018).

An understanding of the contextual sensitivity of IL-2 may guide improved therapeutic bioengineering. For example, initiation of the adverse eosinophilic circuit may be avoided by engineering exogenous IL-2 to recapitulate the quarantining of the B cell zone observed by the primary endogenous sources. Antibody-mediated guidance to T cell zones may achieve this goal, with therapies such as Darleukin (L19-IL-2 fusion) providing proof-of-concept for antibody-guided IL-2 delivery. Alternatively, the competitive advantage of CD8 T cells with self-production of IL-2, even within environments of enriched IL-2 available, suggests that targeting production to CD8 T cells themselves may drive the desired response, for instance in a tumor setting. Here approaches such as in vivo delivery of IL-2 plasmids (Lohr et al., 2001) could be coupled to CD8 T cell–specific promoters. A synthesis of biochemical engineering and context-sensitive design may unlock the long-awaited therapeutic potential of this key immunologic player.

## Materials and methods

### Mice

The *Rosa*^*IL-2*^ allele was generated by inserting the open reading frame of mouse *Il2* (transcript ID ENSMUST00000029275) into the first intron of the Rosa26 locus in C57BL/6N embryonic stem (ES) cells (Schoonjans et al., 2003). The targeting construct consisted, from 5′ to 3′, of (i) 1,082-bp homologous region; (ii) adenovirus major late transcript splice acceptor (Friedrich and Soriano, 1991); (iii) LoxP-flanked neomycin resistance cassette (phosphoglycerate kinase [PGK] promoter−NeoR/KanR−PGK polyadenylation signal); (iv) 3× SV40 polyadenylation signal, kozak-preceded *Il2* open reading frame; (v) IRES sequence; (vi) EGFP sequence; (vii) bovine growth hormone polyadenylation signal; (viii) 4,264-bp homologous region; and finally (ix) the diphtheria toxin subunit A gene (PGK promoter−diptheria toxin A including SV40 small t antigen intron−bovine growth hormone polyadenylation signal) to select against random integration events. The construct was linearized with PvuI. The complete nucleotide sequence of the final targeting vector can be obtained from the authors upon request. Correctly targeted ES cell clones were identified by PCR with primer 5′-TAG​GTA​GGG​GAT​CGG​GAC​TCT-3′ and 5′-GCG​AAG​AGT​TTG​TCC​TCA​ACC-3′; site-specific integration was confirmed by Southern blotting; and ES cells were injected into C57BL/6J albino blastocysts for the generation of chimeric mice. Chimeric males were crossed to C57BL/6J albino for germline transmission and later to a variety of Cre lines on the C57BL/6 background.

*Foxp3*^Thy1.1^ (Liston et al., 2008), *Foxp3*^cre^ (Rubtsov et al., 2008), *Foxp3*^BAC^ (Petzold et al., 2014), *Foxp3*-Tocky (Bending et al., 2018a; Bending et al., 2018b), NKp46^cre^ (Narni-Mancinelli et al., 2011), and CD23^cre^ (Kwon et al., 2008) mice were used on the C57BL/6 background. *Il2*^GFP^ mice (Yui et al., 2001) were purchased from Mutant Mouse Resource and Research Center as stock 009974-MU. *Il2*^cre^ mice stock 029619 (Yamamoto et al., 2013), CD4^cre^ stock 022071, CD8^cre^ stock 008766, *Osx*^cre^ stock 006361, *Cd19*^cre^ stock 006785, *H2*^*dlAb1-Ea*^ (MHCII^−/−^) stock 003584, *CD1d*^*−/−*^ stock 008881, and *B2m*^*−/−*^ stock 002070 mice were purchased from Jackson. *Clec9a*^cre^ (Schraml et al., 2013) mice were kindly provided by Caetano Reis e Sousa (Francis Crick Institute, London, UK). *Il2*^fl/fl^ mice (Popmihajlov et al., 2012) were kindly provided by Michael Farrer (University of Minnesota, Minneapolis, MN). *Kb*^*−/−*^*Db*^*−/−*^ (MHCI^−/−^) mice were kindly provided by Carla Shatz (Stanford University, Stanford, CA). All mice were housed under specific pathogen–free conditions and were fed a standard chow diet, ad libitum. Mice were assessed at 3–18 wk of age with littermate controls, unless stated otherwise. C57BL/6.SJL-*Ptprc*^*a*^/BoyJ (CD45.1) mice were irradiated with 10 Gy over two doses and reconstituted intravenously with 2–5 × 10^6^ total BM cells. Mice were left for ≥7 wk to allow reconstitution before initiating experiments. For neutralization experiments, 3-wk-old littermates were treated with 500 µg anti-IL-5 (BE0198; BioXCell) or isotype control (BE0088; BioXCell) i.p. twice per week for six doses. For Treg transfers, 4 × 10^5^ CD45.1^+^ CD4^+^ Foxp3^Thy1.1+^ Treg and 4 × 10^5^ CD45.2^+^ CD4^+^ Foxp3^Thy1.1−^ CD44^hi^ CD62L^lo^ Tconv cells were cotransferred into *Rag*^*−/−*^ recipients. For other adoptive transfer experiments, CD4^+^CD25^−^ T cells or CD8^+^ T cells were sorted from WT and Rosa^IL-2^ mice and mixed at a 1:1 ratio, and 1 million (CD4^+^ CD25^−^ T cells) or 3.6 million (CD8^+^ T cells) cells were transferred i.p. into congenic recipients. All experiments were performed in accordance with the University of Leuven Animal Ethics Committee guidelines, the Babraham Institute Animal Welfare and Ethics Review Body, or the Animal Care Committee at Maisonneuve-Rosemont Hospital Research Centre. Animal husbandry and experimentation complied with existing European Union and national legislation and local standards or the Canadian Council on Animal Care guidelines. Sample sizes for mouse experiments were chosen in conjunction with the ethics committees to allow for robust sensitivity without excessive use. Mice exhibiting a 20% weight loss or moderate signs of stress (including intermittent hunching, pilo-erection, reduced activity, or poor grooming) were euthanized for ethical reasons.

### Flow cytometry

To obtain single-cell suspensions, spleens and LN were disrupted with glass slides, and bones were crushed with a mortar and pestle, before being filtered through 100-µm mesh. Lung tissue was digested with 0.4 mg/ml Collagenase D (Roche) and 40 mg/ml DNase I (Sigma-Aldrich) prepared in RPMI (Invitrogen) supplemented with 2 mM MgCl_2_, 2 mM CaCl_2_, 20% FBS, and 2 mM Hepes at 37°C for 30 min, followed by filtration through 100-µm mesh. Spleen, BM, and lung subsequently underwent red blood cell lysis. Cells were counted using a Countess cell counter (Thermo Fisher Scientific). Approximately 2 million cells were stained with flow cytometry antibodies. Nonspecific binding was blocked using 2.4G2 supernatant for mouse cells, and dead cells were labeled by fixable viability dye eFluor 780 (Thermo Fisher Scientific). Cells were fixed and permeabilized with 2% formaldehyde or Foxp3 Transcription Factor Staining Buffer Set (eBioscience) according to the manufacturer’s instructions. For cytokine analysis, cells were first stimulated with 500 ng/ml phorbol 12,13-dibutyrate (PdBU), 750 ng/ml ionomycin, and 2 µg/ml Brefeldin A (BrefA; all Tocris Bioscience) in RPMI (Invitrogen) for 4 h at 37°C. For phosphoSTAT staining, cells were fixed with 2% formaldehyde for 30 min, followed by permeabilization with ice-cold 100% methanol for 30 min at 4°C. Cells were stained with anti-phosphoSTAT antibodies in PBS with 2.5% FCS and 2 mM EDTA overnight at room temperature. Flow cytometry samples were acquired on a Yeti/ZE5 (Propel Labs/Bio-Rad), Symphony (BD Biosciences), Fortessa (BD Biosciences), or Aurora (Cytek) spectral flow cytometer. Data was compensated via AutoSpill (Roca et al., 2021a).

### Antibodies

B220 (RA3-6B2), CD122 (TM-b1), CD11b (M1/70), CD127 (A7R34), CD19 (1D3), CD23 (B3B4), CD24 (M1/69), CD25 (PC61), CD3 (145-2C11), CD4 (RM4-5), CD44 (IM7), CD45.1 (A20), CD45.2 (104), CD62L (MEL-14), CD69 (H1.2F3), CD8a (53-6.7), F4/80 (BM8), Foxp3 (FJK-16s), GATA-3 (L50-823), Helios (22F6), IgM (Il/41), IL-2 (JES6-5H4), IRF4 (3E4), MHCII (I-A/I-E; M5/114.15.2), Neuropilin (CD304; 3DS304M), pSTAT5 (SRBCZX), RORgT (AFKJS-9), ST2 (IL-33R; RMST2-2), TCR γ/δ (GL3), and TCR-β (H57-597) antibodies were from Thermo Fisher Scientific. Bcl-6 (K112-91), CCR2 (475301), CCR9 (CW-1.2), CD103 (M290), CD11b (M1/70), CD117/c-kit (2B8), CD127 (SB/199), CD19 (1D3), CD21/35 (7G6), CD25 (PC61), CD4 (GK1.5), CD45 (30-F11), CD62L (MEL-14), CD80 (16-10A1), CD8a (53-6.7), CD95 (Jo2), FR4 (12A4), GATA-3 (L50-823), GITR (DTA-1), IgD (11-26c.2a), IgE (R35-72), Ly6C (AL-21), NK1.1 (PK136), PDCA-1 (927), RORgT (Q31-378), Siglec F (E50-2440), and TCR-β (H57-597) antibodies were from BD Biosciences. CCR3 (J073E5), CD11c (N418), CD103 (2E7), CD122 (TM-b1), CD132 (Tugm2), CD138 (281-2), CD25 (PC61), CD3 (145-2C11), CD3 (17A2), CD4 (RM4-5), CD44 (IM7), CD45 (30-F11), CD45.1 (A20), CD45.2 (104), CD64 (X54-5/7.1), CD8a (53-6.7), CD90.1/Thy1.1 (HIS51), CD90.2 (53-2.1), CXCR5 (L138D7), F4/80 (BM8), GFP (FM264G), Helios (22F6), ICOS (C398.4A), IFNg (XMG1.2), IL-5 (TRFK5), Ki-67 (16A8), KLRG1 (2F1/KLRG1), Ly6C (HK1.4), Ly6G (1A8), Ly6G&Ly6C (Gr-1; RB6-85C), MHCII (I-A/I-E; M5/114.15.2), NK1.1 (PK136), NKp46 (29A1.4), PD-1 (29F.1A12), PDCA-1 (927), pSTAT3 (13A3-1), T-bet (4B10), TNFRII (TR75-89), and XCR1 (ZET mouse IgG2b) antibodies were from BioLegend. CCR3 (83101) and CD11c (N418) antibodies were from R&D Systems. CD25 (PC61) was from Bio-Rad, and Eomes (REA116), Foxp3 (REA788), and T-bet (4B10) antibodies were from Miltenyi Biotec.

### In vitro cytokine stimulation

Splenocytes were processed as described above and resuspended in complete RPMI (Invitrogen). Cells were stimulated with 100 ng/ml IL-2, IL-7, or IL-15 for 25 min at 37°C. Cells were immediately fixed with 2% formaldehyde for phosphoSTAT staining.

### In vitro suppression assay

CD4^+^ Tregs (CD4^+^CD25^+^) and CD8^+^ Tregs (CD8^+^CD25^+^CD103^+^) from *Cd19*^cre^*Rosa*^*IL-2*^ and littermate mice and Tconv cells (Thy1.1^−^CD44^lo^CD62L^hi^) from *Foxp3*^*Thy1*.*1*^ mice were isolated from spleens and LN by negative selection with MagniSort Streptavidin Negative Selection Beads (Thermo Fisher Scientific) followed by cell sorting (BD FACSAria III). Antigen-presenting cells were sourced by digesting *Rag*^*−/−*^ spleens. For the suppression assay, 1 × 10^5^ Tregs were plated per well in complete RPMI along with 5 × 10^4^ *Rag*^*−*/−^ splenocytes preincubated with 1 µg/ml anti-CD3 (Thermo Fisher Scientific). CD4^+^ and CD8^+^ Tconv cells (responders) were labeled with CellTrace Violet (Thermo Fisher Scientific), plated at the indicated ratios with Tregs, and incubated for 3 d at 37°C. Suppression was calculated by comparing the proliferative index of the sample with the proliferative index of Tconv cells cultured in identical conditions except without cocultured Tregs.

### Cytokine analysis

To measure IL-2 in spleen tissue, samples (5 mg) were placed in Protein Quant Sample Lysis buffer containing Protein Inhibitor Cocktail (Thermo Fisher Scientific). The tissues were homogenized in a FastPrep instrument (MP Biomedicals) with Lysing Matrix D and then incubated on a shaker for 20 min at 4°C. The lysate obtained was centrifuged at 16,000 *g* for 1 min at 4°C. IL-2 levels in lysate, supernatant, or mouse serum were then measured with the ProQuantum High-Sensitivity mouse IL-2 Immunoassay (Thermo Fisher Scientific) according to the manufacturer’s instructions. Serum samples were analyzed for IL-5 and IL-13 levels using ProCartaPlex Immunoassays (Thermo Fisher Scientific) and acquired on the Luminex Bio-Plex 3D Suspension Array System (Bio-Rad).

### Treg expression of IL-2 in vitro

CD4^+^ Treg and Tconv cells were sorted from Foxp3Thy1.1 mice using CD4-eFluor450, fixable viability dye eFluor780, and Thy1.1-PE to >99% purity. T cells (1.5 × 10^5^ per well) were plated on U-bottom 96-well plates precoated with varying concentrations of anti-CD3 (clone 145-2C11). Anti-CD28 (clone 37.51) and anti-CD25 (clone PC61) were added in solution at 5 and 10 μg/ml, respectively, along with IL-7 (100 ng/ml). Cells were cultured for 72 h, at which time the supernatant was analyzed with the ProQuantum assay as above and the cell pellet by quantitative PCR.

To measure *Il2* transcript, RNA extraction from cell pellets was performed using the RNeasy Micro Kit (Qiagen) according to the manufacturer’s protocol. cDNA was synthesized using iScript cDNA Synthesis Kit (Bio-Rad) according to the manufacturer’s protocol. Real-time quantitative PCR was performed in QuantStudio 1 Real-Time PCR System (Applied Biosystems). The initial step was 10-min incubation at 95°C, and the template was amplified for 40 cycles of 30-s incubation at 95°C, 30-s incubation at 55°C, and 30-s incubation at 72°C. All data were normalized to the PPIA reference gene control. Primers for *Ppia* were 5′-TTC​ACC​TTC​CCA​AAG​ACC​AC-3′ and 5′-CAA​ACA​CAA​ACG​GTT​CCC​AG-3′. Primers for *Il2* were 5′-GCG​GCA​TGT​TCT​GGA​TTT​GAC​TC-3′ and 5′-CCA​CCA​CAG​TTG​CTG​ACT​CAT​C-3′.

### Immunofluorescence

LNs and spleen were embedded in optimal cutting temperature compound (OCT CryoMatrix; Thermo Fisher Scientific) and snap frozen in the vapor phase of liquid nitrogen. 10-µm (LN) or 20-µm (spleen) sections were generated on a Leica CM1850 Cryostat. For pSTAT5 imaging, spleen sections were fixed in 2% paraformaldehyde overnight at 4°C and cryoprotected in 30% sucrose (#S0389; Sigma-Aldrich) overnight at 4°C, followed by incubation with 2% Triton X-100 for 30 min and blocking with 5% BSA for 1 h at room temperature. Sections were stained overnight with anti-B220-biot (1:200, RA3-6B2; Thermo Fisher Scientific), anti-CD3-Alexa Fluor 647 (1:500, 500A2; BioLegend), and anti-Phospho-Stat5 (Tyr694; 1:50, C11C5; Cell Signaling Technology). The next day, streptavidin Alexa Fluor 790 (1:1,000; Invitrogen), anti-rabbit Alexa Fluor 555 (1:1,000; Thermo Fisher Scientific), and DAPI (1:1,000, Life Technologies) were incubated for 30 min. Slides were washed and mounted with Fluormount-G. Confocal images were obtained by using a single-plane confocal microscopy on a Leica Stellaris 8 system with 20× and 63× Objectives. For CD8 Foxp3 imaging, lymph node sections were fixed in 2% paraformaldehyde, followed by incubation with 2% Triton X-100 for 30 min and blocking with 5% BSA for 1 h at room temperature. Sections were stained overnight with anti-CD8α-eFluor 450 (1:20, 48-0081-82; eBioscience), anti-IgD-Alexa Fluor 594 (1:400, 405740; BioLegend), and anti-Foxp3-Alexa Fluor 488 (1:200, 53-5773-82; Thermo Fisher Scientific) and mounted with Fluormount-G. Confocal images were obtained by using a single-plane confocal microscopy on a Zeiss LSM 780 system with 20× objective.

### Image analysis

For analysis of cellular pStat5 levels across the spleen, an image analysis pipeline was constructed using Fiji (Schindelin et al., 2012) and CellProfiler (Stirling et al., 2021). Images analyzed were single z-slices of a large overview image of spleen cryosections labeled with a DAPI nuclear counterstain to visualize all cells present in the section and labeled for CD3 and B220 to allow for classification into T or B cells, respectively. A pStat5 antibody was used to assess nuclear levels of this transcription factor. After adding two additional image channels containing segmentation masks for white pulp and T and B cell zones, the overview images were subdivided into a collection grid consisting of 630 × 630-pixel image tiles, with a 30-pixel overlap between neighboring tiles.

Individual tiles were analyzed in CellProfiler. In brief, the analysis pipeline includes the RunCellpose module (Stringer et al., 2021) to segment a nuclear mask based on the DAPI channel. Cell masks are constructed by dilation of the nuclear mask. Measurements extracted for each segmented cell and nucleus included size, morphology, intensity (for each of the six image channels, i.e., DAPI, CD3, B220, pStat5, WhitePulp mask, and TZone mask), and location, including the x and y coordinates within the image tile, as well as any overlap with white pulp, red pulp, T cell zone, or B cell zone. Data for all object populations was exported in CSV format and further processed in R.

For CD8 Foxp3 imaging, images were processed in ImageJ (https://imagej.nih.gov/ij/download.html). The total CD8^+^ cells within the B follicle (IgD^+^ area) were quantified using the multipoint selection tool when a red or dark center (nucleus) surrounded by CD8 surface staining could be identified. Then, Foxp3^+^ among CD8^+^ cells within the B follicle area were quantified.

### Statistics

All statistical analysis was performed using GraphPad Prism or R. Comparisons between groups were performed using paired or unpaired two-tailed Student’s *t* tests, one-way ANOVA, or two-way ANOVA as appropriate. When required, post hoc Sidak’s or Tukey’s multiple comparison tests were performed. Survival data were analyzed using Mantel–Cox log-rank test. Nonparametric testing was performed when data were not normally distributed. FlowSOM, t-distributed stochastic neighbor embedding (tSNE), heatmap analysis, and principal component analysis were performed in R (v3.6.2) using in-house scripts (Roca et al., 2021b). For tSNE, datasets were analyzed with 10,000 iterations and perplexity of 30. Comparison of tSNE plots and dendrograms was performed in R using in-house scripts (Pasciuto et al., 2020). Values are represented as mean ± SEM, unless otherwise indicated.

### Supplemental material

Fig. S1 shows thymic changes in CD8^cre^ Rosa^IL-2^ and CD4^cre^ Rosa^IL-2^ mice and differential protein expression in T cell splenocyte clusters. Fig. S2 shows differential protein expression in splenocyte clusters from *Foxp3*^cre^*Rosa*^*IL-2*^ mice. Fig. S3 shows cell quantification, correlations, and differential protein expression in mixed WT/CD4^cre^ Rosa^IL-2^ BM chimeras. Fig. S4 shows cell quantification, correlations, and cytokine analysis in *Cd19*^cre^ Rosa^IL-2^, CD23^cre^ Rosa^IL-2^, and *Osx*^cre^ Rosa^IL-2^ mice. Fig. S5 shows CD8 Foxp3^+^ gating strategy, reporter protein expression, and FlowSOM analysis, in addition to cell quantification in MHC KO strains.

## Data Availability

The datasets generated for Fig. 1 are available in FlowRepository (FR-FCM-Z443, FR-FCM-Z448, FR-FCM-Z444, FR-FCM-Z44V). Other datasets that support the findings of this study are available from the corresponding author upon reasonable request.
