# Correction: Context-dependent effects of IL-2 rewire immunity into distinct cellular circuits

**DOI:** 10.1084/jem.2021239107142022c

**Published:** 2022-07-25

**Authors:** Carly E. Whyte, Kailash Singh, Oliver T. Burton, Meryem Aloulou, Lubna Kouser, Rafael Valente Veiga, Amy Dashwood, Hanneke Okkenhaug, Samira Benadda, Alena Moudra, Orian Bricard, Stephanie Lienart, Pascal Bielefeld, Carlos P. Roca, Francisco José Naranjo-Galindo, Félix Lombard-Vadnais, Steffie Junius, David Bending, Masahiro Ono, Tino Hochepied, Timotheus Y.F. Halim, Susan Schlenner, Sylvie Lesage, James Dooley, Adrian Liston

Vol. 219, No. 7 | 10.1084/jem.20212391 | June 14, 2022

The authors regret that Masahiro Ono was inadvertently omitted from the author list in the original version of their article. The corrected author list appears above.

The error appears in print and in PDFs downloaded before July 14, 2022.

